# An Improved Spider Wasp Optimizer for UAV Three-Dimensional Path Planning

**DOI:** 10.3390/biomimetics9120765

**Published:** 2024-12-16

**Authors:** Haijun Liang, Wenhai Hu, Lifei Wang, Ke Gong, Yuxi Qian, Longchao Li

**Affiliations:** Air Traffic Management Institute, Civil Aviation Flight University of China, Deyang 618307, China

**Keywords:** unmanned aircraft, path planning, Spider Wasp Optimizer (SWO), Improved Spider Wasp Optimizer (ISWO), terrain mapping, mathematical model, optimal path

## Abstract

This paper proposes an Improved Spider Wasp Optimizer (ISWO) to address inaccuracies in calculating the population (N) during iterations of the SWO algorithm. By innovating the population iteration formula and integrating the advantages of Differential Evolution and the Crayfish Optimization Algorithm, along with introducing an opposition-based learning strategy, ISWO accelerates convergence. The adaptive parameters trade-off probability (TR) and crossover probability (Cr) are dynamically updated to balance the exploration and exploitation phases. In each generation, ISWO optimizes individual positions using Lévy flights, DE’s mutation, and crossover operations, and COA’s adaptive update mechanisms. The OBL strategy is applied every 10 generations to enhance population diversity. As the iterations progress, the population size gradually decreases, ultimately yielding the optimal solution and recording the convergence process. The algorithm’s performance is tested using the 2017 test set, modeling a mountainous environment with a Gaussian function model. Under constraint conditions, the objective function is updated to establish a mathematical model for UAV flight. The minimal cost for obstacle-avoiding flight within the specified airspace is obtained using the fitness function, and the flight path is smoothed through cubic spline interpolation. Overall, ISWO generates high-quality, smooth paths with fewer iterations, overcoming premature convergence and the insufficient local search capabilities of traditional genetic algorithms, adapting to complex terrains, and providing an efficient and reliable solution.

## 1. Introduction

**Figure 1:**
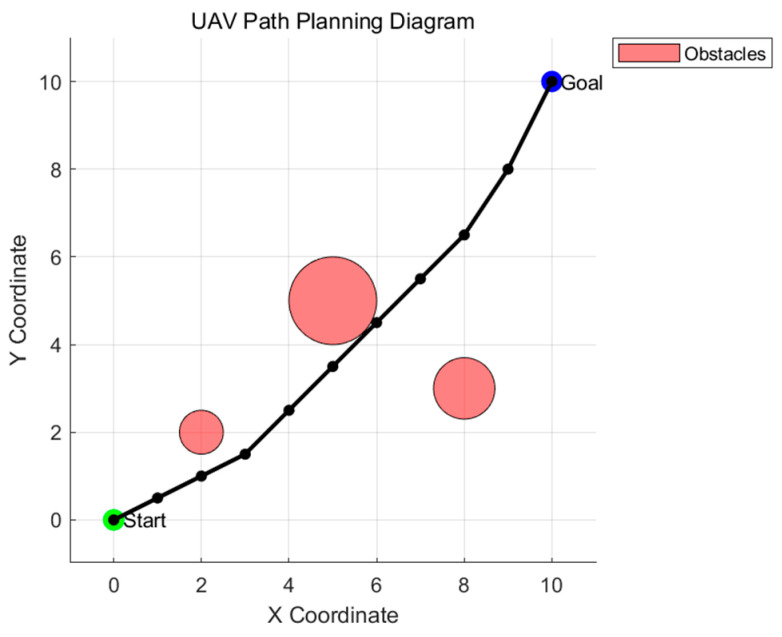
Simplified diagram of UAV path planning.

Amid the relentless advancement of technology and the escalating demands of human production and daily life, small unmanned aerial vehicles (UAVs) have garnered significant attention due to their myriad advantages—such as flexible configurations, compact dimensions, and robust adaptability. Currently, they are extensively employed in reconnaissance and surveillance [1], urban logistics [2], power grid inspections [3], firefighting and rescue operations [4], and urban patrolling [5], among numerous other domains. These attributes empower UAVs not only to effectively assist humans but even to entirely supplant human involvement in various scenarios, including tracking, exploration, transportation, and military missions [6]. In UAV technology, path planning serves as the cornerstone for achieving UAV autonomy. It furnishes the essential technical support to ensure that UAVs can navigate safely and efficiently from the starting point to the destination. Figure 1 below illustrates the simplified process of UAV flight path planning. The pink areas represent obstacles. The UAV must navigate from the starting point to the endpoint while avoiding these pink obstacles.

In recent years, research on path planning has been broadly categorized into three types based on solution strategies. The first category comprises population-based algorithms, such as the, newly introduced in 2024, Hippopotamus Optimization Algorithm (HO) [7] and Black-winged Kite Algorithm (BKA) [8], as well as recent developments like the Dung Beetle Optimizer (DBO) [9], Lemurs Optimizer (LO) [10], Crayfish Optimization Algorithm (COA) [11], and earlier methods like Particle Swarm Optimization (PSO) [12].

The second category involves physics-based algorithms, such as the Kepler Optimization Algorithm (KOA) [13], Light Spectrum Optimizer (LSO) [14], and the Sine Cosine Algorithm (SCA) [15]. There are also human-inspired metaheuristic algorithms, including the Gold Rush Optimizer (GRO) [16] and the Catch Fish Optimization Algorithm (CFOA) [17]. Based on the simulation experiments of path planning described below, it was discovered that the CFOA algorithm, newly proposed this year, exhibits inefficiency in engineering applications. Additionally, it demonstrates low stability and reliability on the CEC2017 [18] test suite. The HO and BKA algorithms, when tested on benchmark functions, display higher maximum, average, and minimum values across multiple test functions, indicating a propensity to become trapped in local optima and a lack of global search capability. The COA algorithm lacks robust global exploration ability and is particularly prone to local optima during the later stages of optimization. The DBO algorithm may suffer from insufficient search efficiency in high-dimensional and complex problems, leading to slower convergence rates. The LO algorithm requires improvements in search efficiency when applied to complex three-dimensional UAV path planning. The PSO algorithm tends to converge prematurely during the early stages of the search, potentially missing the global optimal solution. The SCA algorithm exhibits periodicity in its search process, making it susceptible to the influence of local optima and reducing its ability to dynamically adapt during the search. The GRO algorithm may prematurely converge to local optima when individual behaviors become overly homogeneous, thus limiting global exploration. This paper introduces a new nature-inspired metaheuristic algorithm, the Spider Wasp Optimizer (SWO) [19], which incorporates multiple unique update strategies and is applicable to various optimization problems with differing exploration and exploitation requirements. The effectiveness of SWO was evaluated using several mathematical benchmarks and real-world optimization problems. Although SWO offers advantages in UAV path planning, such as efficient global search capabilities and strong convergence performance, it simulates relatively complex biological behaviors, resulting in high computational complexity. When addressing high-dimensional path planning problems, it may consume substantial computational resources, which is disadvantageous for resource-constrained embedded UAV systems. Therefore, during the iterative process, an Improved Spider Wasp Optimizer (ISWO) updates the positions of search agents through hunting and nesting behaviors (exploration phase) and mating behaviors (exploitation phase). In the hunting and nesting behaviors, strategies like dynamic parameter adjustment and Lévy flights [20] are employed to enhance the exploration ability of the solutions. Differential Evolution (DE) [21] generates new candidate solutions through mutation and crossover operations during the mating phase, maintaining population diversity and promoting search efficiency. COA optimizes individual positions during the mating phase by combining the foraging and avoidance mechanisms of COA, enhancing local search capability. Furthermore, the algorithm re-applies the opposition-based learning [22] strategy that generates opposite solutions periodically during initialization and iterations, expanding the search space and enhancing global search capability. To reduce computational load and accelerate convergence, the algorithm adopts a population size reduction strategy, gradually decreasing the number of search agents. Experimental results indicate that the Improved Spider Wasp Optimizer exhibits faster convergence rates and higher solution quality when solving complex optimization problems, validating the importance of integrating multiple optimization strategies and opposition-based learning. Moreover, the ISWO algorithm achieved the best performance on the CEC2017 test suite.

## 2. The Spider Wasp Optimizer

**Figure 2:**
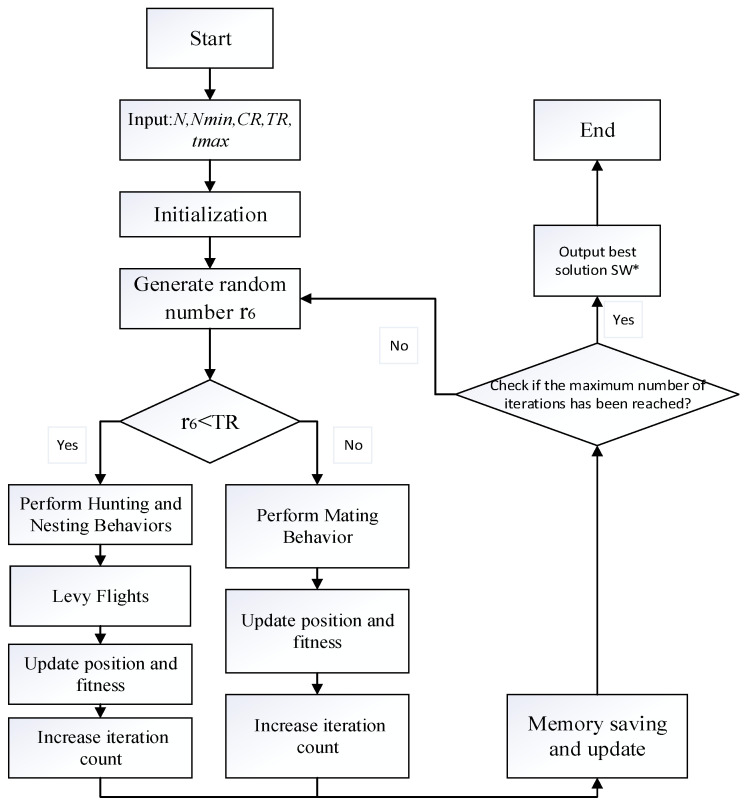
Flowchart of the SWO algorithm.

The Spider Wasp Optimizer (SWO) is a metaheuristic optimization algorithm inspired by the behaviors of spider wasps. This algorithm emulates the trade-offs between hunting, nesting, and mating behaviors exhibited by spider wasps and applies these mechanisms to global optimization problems. Initially, the algorithm performs the initialization of all search agents (spider wasps). The algorithm’s inputs include the number of search agents, the maximum number of iterations, the upper and lower bounds of the search space, the dimensionality of the problem, and the objective function. After the initialization phase is concluded, the algorithm evaluates the fitness of each spider wasp’s initial position by invoking the objective function for each agent. If a spider wasp’s fitness value exceeds the current optimal fitness, the best solution and corresponding fitness are updated accordingly. While the condition $t<t_{max}$ holds, the main loop continues execution until the maximum number of iterations is reached. During each iteration, the algorithm dynamically adjusts specific parameters. If a randomly generated number rand is less than the trade-off probability TR, the algorithm elects to perform the hunting and nesting behaviors, updating the positions of each spider wasp accordingly. Conversely, if rand≥TR, the mating behavior is enacted. In the mating phase, the algorithm generates a new individual by computing the positional differences between the current solution and other wasps. The new individual’s position is obtained by calculating the difference between two positions and updating the position using the crossover probability Cr. If the newly generated individual yields superior performance compared to the current one, its position is updated and its fitness is recalculated. If it does not improve upon the current solution, the algorithm reverts to the prior optimal position. At the conclusion of each iteration, the population size of the spider wasps is decremented in accordance with Equation (25). This progressive reduction in the number of search agents during the later iterations serves to accelerate the convergence rate. Upon reaching the maximum iteration count $t_{max}$, the algorithm terminates, outputting the optimal solution and its associated fitness value. Simultaneously, the convergence curve records the best fitness value at each iteration, facilitating analysis of the algorithm’s convergence characteristics. The detailed algorithmic workflow is illustrated in Figure 2.

### 2.1. The Generation of the Initial Population

In the proposed algorithm, each spider wasp (female wasp) embodies a solution of the current generation and can be encoded within a d-dimensional vector using the following expression:

(1) $$\vec{SW}=\left[\begin{array}{c} x_{1},x_{2},x_{3},\ldots ,x_{D} \end{array}\right]$$

A set of N vectors can be randomly generated between the pre-specified upper initial parameter boundary vector $\vec{H}$ and the lower initial parameter boundary vector $\vec{L}$, as detailed below:

(2) $$SW_{Pop}=\left[\begin{array}{cccc} {\mathrm{sw}}_{1,1} & {\mathrm{sw}}_{1,2} & \ldots & {\mathrm{sw}}_{1,D}\\ {\mathrm{sw}}_{2,1} & {\mathrm{sw}}_{2,2} & \ldots & {\mathrm{sw}}_{2,D}\\ \vdots & \vdots & \vdots & \vdots \\ {\mathrm{sw}}_{N,1} & {\mathrm{sw}}_{N,2} & \ldots & {\mathrm{sw}}_{N,D} \end{array}\right]$$

Here, ${SW}_{pop}$ represents the initial population of spider wasps. The following equation can be employed to randomly generate any solution within the search space:

(3) $${\bar{SW}}_{i}^{t}=\vec{L}+\vec{r}\times \left(\vec{H}-\vec{L}\right)$$

Here, t denotes the generation index, and i represents the population index (i=1,2,…,N). The vector $\vec{r}$ is a d-dimensional vector, whose elements are randomly initialized values between 0 and 1. Subsequently, the mathematical simulation of spider wasp behaviors is introduced to establish a novel metaheuristic algorithm for solving optimization problems. These behaviors are as follows:

Hunting and nesting behaviors.

Mating behaviors.

### 2.2. Search Phase (Exploration)

This phase simulates the behavior of female wasps searching for the most suitable spiders to feed their larvae. During this stage, female wasps conduct random exploration within the search space using a fixed step size, as previously described, to locate spiders that are optimal for their offspring. This behavior is modeled using the method illustrated in Equation (4), which updates each female wasp’s current position with constant motion in each generation t, thereby emulating the exploratory behavior of female wasps.

(4) $${\bar{SW}}_{i}^{t+1}={\bar{SW}}_{i}^{t}+\mu_{1}*\left({\bar{SW}}_{a}^{t}-{\bar{SW}}_{b}^{t}\right)$$

Here, a and b are two indices randomly selected from the population to determine the exploration direction of the female wasp. The parameter $\mu_{1}$ is utilized to establish the constant motion based on the current direction, employing the following formula:

(5) $$\mu_{1}=|rn|*r_{1}$$

Here, $r_{1}$ is a randomly generated value within the interval [0, 1], and $r_{n}$ is another random number, though it is generated using a normal distribution. Female wasps occasionally lose track of dropped spiders within tennis webs, prompting them to search the entire vicinity surrounding the exact drop location of the spider. Based on this behavior, an alternative equation with a distinct exploration method has been formulated to enable the proposed algorithm to explore the area around the dropped spider, with a smaller step size than Equation (4). This equation similarly updates the current position of each female wasp through constant motion in each generation, where the position representing the dropped spider’s location is embodied by a female wasp randomly selected from the population. The equation is described as follows:

(6) $${\bar{SW}}_{i}^{t+1}={\bar{SW}}_{c}^{t}+\mu_{2}*\left(\vec{L}+\vec{r_{2}}*\left(\vec{H}-\vec{L}\right)\right)$$

(7) $$\mu_{2}=B*\cos(2\pi l)$$

(8) $$B=\frac{1}{1+e^{l}}$$

Here, c is an index randomly selected from the population, and l is a randomly generated value between 1 and −2. Equations (4) and (6) complement each other in exploring the search space and identifying the most promising regions. Finally, as detailed below, when generating the next position of a female wasp, a random selection is made between Equations (4) and (6):

(9) $${\vec{SW}}_{i}^{t+1}=\left\{\begin{array}{c} Eq.(4)\;\;\;\;r_{3}<r_{4},\\ Eq.(6)\;\;\;\;otherwise, \end{array}\right.$$

Here, $r_{3}$ and $r_{4}$ are two randomly generated numbers within the interval [0, 1].

### 2.3. Tracking and Evasion Phase (Exploration and Exploitation)

Upon locating prey, the spider wasps endeavor to attack the prey at the center of the web; however, the prey may descend to the ground in an attempt to escape. The wasps subsequently track these fallen spiders, paralyze them, and transport them to pre-prepared nests. In certain instances, the wasps lose track of spiders that have fallen from the web’s center, indicating that while the wasps are attempting to capture the spiders, the spiders are simultaneously evading capture. This behavior emulates two distinct tendencies: the first involves wasps hunting spiders to secure them, wherein Equation (10) is employed to update the positions of the spider wasps for tracking the prey; the second tendency simulates the increasing of the distance between them through a designed distance factor, allowing the prey to escape and conceal themselves in regions distant from the wasps as the current iteration count progresses.

(10) $${\vec{SW}}_{i}^{t+1}={\vec{SW}}_{i}^{t}+C*\left|2*\vec{r_{5}}*{\vec{SW}}_{a}^{t}-{\vec{SW}}_{i}^{t}\right|$$

(11) $$C=\left(2-2*\left(\frac{t}{t_{max}}\right)\right)*r_{6}$$

Here, a is an index randomly selected from the population; t and $t_{max}$ denote the current and maximum evaluation counts, respectively; $\vec{r_{5}}$ is a vector of values randomly generated within the interval [0, 1]; and $r_{6}$ is a randomly generated number within the interval [0, 1]. When a spider escapes from a female wasp, the distance between the female wasp and the spider gradually increases. This phase initially represents the exploitation stage, and as the distance increases, exploitation progressively transitions into exploration. This behavior is modeled by the following equation:

(12) $${\vec{SW}}_{i}^{t+1}={\vec{SW}}_{i}^{t}*\vec{\nu c}$$

Here, $\vec{v_{c}}$ is a vector generated from a normal distribution within the interval [−k,k]. Consequently, k is determined using Equation (13) to progressively increase the distance between the female wasp and the spider.

(13) $$k=1-\left(\frac{t}{t_{max}}\right)$$

The trade-off between these two tendencies is implemented randomly, as illustrated by the following equation:

(14) $${\vec{SW}}_{i}^{t+1}=\left\{\begin{array}{c} Eq.(10)\;r_{3}<r_{4}\\ Eq.(12)\;otherwise \end{array}\right.$$

At the commencement of the optimization process, all wasps employ an exploration mechanism to conduct a global search of the optimization problem’s domain, aiming to identify promising regions that may harbor near-optimal solutions. Throughout the iterative process, the algorithm leverages tracking and evasion mechanisms to explore and exploit the areas surrounding the current wasps, thereby mitigating the risk of becoming trapped in local minima. Finally, the transition between the search phase and the tracking mechanism is regulated according to the following equation:

(15) $${\vec{SW}}_{i}^{t+1}=\left\{\begin{array}{c} Eq.(9)\;p<k\\ Eq.(14)\;otherwise \end{array}\right.$$

**Figure 3:**
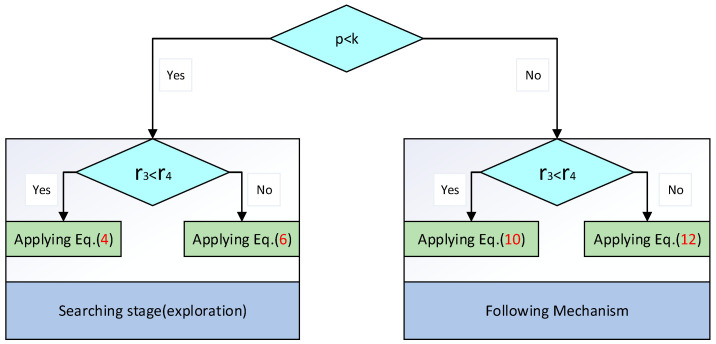
Trade-off between the search phase and the subsequent tracking and evasion mechanisms.

Here, p is a randomly generated number within the interval [0, 1]. The trade-off between the search and tracking mechanisms is illustrated in Figure 3.

### 2.4. Nesting Behavior (Exploitation Phase)

Female wasps transport paralyzed spiders into pre-prepared nests and engage in various nesting behaviors, such as excavating in the soil, constructing mud nests, or utilizing existing cavities. In the proposed algorithm, we employ two equations to simulate these nesting behaviors. The first equation models the transportation of spiders into the most suitable nesting areas, facilitating the placement of paralyzed spiders and the oviposition of eggs on their abdomens.

(16) $${\vec{SW}}_{i}^{t+1}={\vec{SW}}^{*}+\cos(2\pi l)*\left({\vec{SW}}^{*}-{\vec{SW}}_{i}^{t}\right)$$

Here, ${SW}^{*}$ represents the best solution obtained to date. The second equation constructs nests at the positions of female wasps randomly selected from the population, utilizing an additional step size to prevent the construction of two nests at the same location. The equation is designed as follows:

(17) $${\bar{SW}}_{i}^{t+1}={\bar{SW}}_{a}^{t}+r_{3}*|\gamma |*\left({\bar{SW}}_{a}^{t}-{\bar{SW}}_{i}^{t}\right)+\left(1-r_{3}\right)*\vec{U}*\left({\bar{SW}}_{b}^{t}-{\bar{SW}}_{c}^{t}\right)$$

Here, $r_{3}$ is a randomly generated number within the interval [0, 1]; γ is a value generated based on Lévy flight; a,b, and c are indices of three solutions randomly selected from the population; $\vec{U}$ is a binary vector used to determine when to apply a step size in order to prevent the construction of two nests at the same location. The binary vector $\vec{U}$ is assigned according to the following formula:

(18) $$\vec{U}=\left\{\begin{array}{c} 1\;\vec{r_{4}}>\vec{r_{5}}\\ 0\;otherwise \end{array}\right.$$

Here, $\vec{r_{4}}$ and $\vec{r_{5}}$ are two vectors representing randomly generated values within the interval [0, 1]. Equations (16) and (17) are randomly swapped according to the following formula:

(19) $${\bar{SW}}_{i}^{t+1}=\left\{\begin{array}{c} Eq.(16)\;r_{3}<r_{4}\\ Eq.(17)\;otherwise \end{array}\right.$$

**Figure 4:**
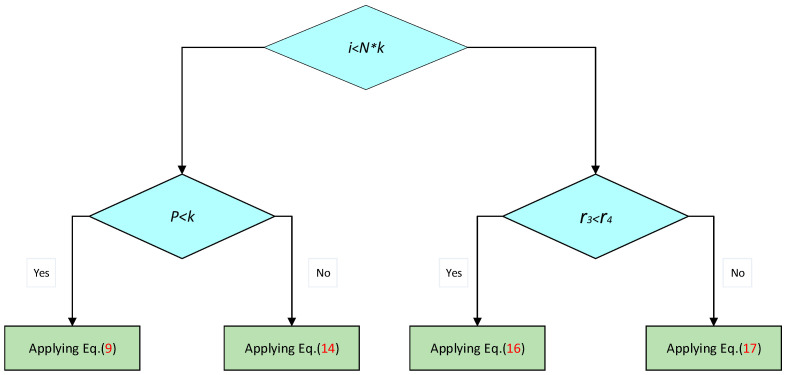
Flowchart of hunting and nesting behaviors in SWO.

Finally, the trade-off between hunting and nesting behaviors is achieved through Equation (20), as illustrated in Figure 4. At the onset of the optimization process, all spider wasps search for their respective spiders. Subsequently, the wasps guide suitable individuals to the pre-prepared nests.

(20) $${\bar{SW}}_{i}^{t+1}=\left\{\begin{array}{c} Eq.(15)\;i<N*k\\ Eq.(19)\;otherwise \end{array}\right.$$

### 2.5. Mating Behavior

Each spider wasp embodies a candidate solution within the current generation, while the eggs of the spider wasps signify newly synthesized potential solutions for that generation. New solutions/spider wasp eggs are generated according to the following equation:

(21) $$SW_{i}^{t+1}=Crossover(SW_{i}^{t},SW_{m}^{t},\mathrm{CR})$$

Here, Crossover denotes the uniform crossover operator applied between solutions ${SW}_{m}^{t}$ and ${SW}_{i}^{t}$, and the crossover probability is referred to as the crossover rate (CR). ${SW}_{m}^{t}$ and ${SW}_{i}^{t}$ represent the vectors of male and female spider wasps, respectively. In the proposed algorithm, male spider wasps are generated according to the following formula to differentiate them from female wasps:

(22) $${\bar{SW}}_{m}^{t+1}={\bar{SW}}_{i}^{t}+e^{l}*|\beta |*{\vec{v}}_{1}+\left(1-e^{l}\right)*|\beta_{1}|*{\vec{v}}_{2}$$

Here, β and $\beta_{1}$ are two numbers randomly generated based on a normal distribution, e is the natural constant, and $\vec{v_{1}}$ and $\vec{v_{2}}$ are generated according to the following formula:

(23) $${\vec{v}}_{1}=\left\{\begin{array}{c} {\vec{x}}_{a}-{\vec{x}}_{i}f({\vec{x}}_{a})<f({\vec{x}}_{i})\\ {\vec{x}}_{i}-{\vec{x}}_{a}\;otherwise \end{array}\right.$$

(24) $${\vec{v}}_{2}=\left\{\begin{array}{c} {\vec{x}}_{b}-{\vec{x}}_{c}f({\vec{x}}_{b})<f({\vec{x}}_{c})\\ {\vec{x}}_{c}-{\vec{x}}_{b}\;otherwise \end{array}\right.$$

Here, a, b, and c are indices of three solutions randomly selected from the population, with a≠i≠b≠ *c*. The crossover operation is employed to recombine the genetic material of two parent spider wasps, thereby generating an offspring (egg) that simultaneously possesses characteristics from both parents. The trade-off between hunting and mating behaviors is governed by a predefined factor known as the behavioral weight (TR).

### 2.6. Population Reduction and Memory Saving

During the iterative process, certain wasps within the population are terminated to allocate additional function evaluations to other wasps, thereby reducing population diversity and accelerating convergence toward near-optimal solutions. In each comprehensive function evaluation, the size of the new population is updated according to the following equation:

(25) $$N=N_{min}+(N-N_{min})\times k$$

Here, $N_{min}$ denotes the minimum population size employed to prevent the optimization process from becoming trapped in local minima at various stages. As the number of iterations increases, k linearly decreases from 1 to 0. Finally, our proposed algorithm achieves memory efficiency by storing the optimal spider positions acquired by each wasp, facilitating updates in subsequent generations (Algorithm 1).
**Algorithm 1** The proposed SWOInput:$N,\;Nmin,\;\mathrm{CR},\;\mathrm{TR},\;t_{max}$Output:$\vec{{SW}^{*}}$1.Initialize N $\mathrm{female}\;\mathrm{wasps},\;\vec{{SW}_{i}}$ (i=1, 2, ..., N), using Equation (3)2.$\mathrm{Evaluate}\;\mathrm{each}\;\vec{{SW}_{i}}$ $\mathrm{and}\;\mathrm{finding}\;\mathrm{the}\;\mathrm{one}\;\mathrm{with}\;\mathrm{the}\;\mathrm{best}\;\mathrm{fitness}\;\mathrm{in}\;\vec{{SW}^{*}}$3.*t* = 1; //the current function evaluation4.$\mathrm{while}\;(t\;<\;t_{max}$)5.  $r_{6}$: generating a random number between 0 and 16.  $\mathrm{if}\;(r_{6}<\mathrm{TR}$) %% Hunting and Nesting behaviors7.    for i=1:N
8.    *Applying*
Figure 2
9.    *Compute*
$f(\vec{{SW}_{i}}$)10.    *t* = *t* + 1;11.   End for12.  Else %% Mating Behavior13.   for i=1:N
14.   *Applying* Equation (21)15.   *t* = *t* + 1;16.   End for17.  End if18.  Applying *Memory Saving*19.  Updating *N* using Equation (25)20.End while

## 3. Improved Spider Wasp Optimizer

The Improved Spider Wasp Optimizer (ISWO) synergistically combines the advantages of the Spider Wasp Optimizer (SWO), the Crayfish Optimization Algorithm (COA), and Differential Evolution (DE). It refines the original Equation (25), adaptively adjusts the parameters TR [23] and Cr [24], and introduces the opposition-based learning (OBL) strategy to expedite convergence.

**Figure 5:**
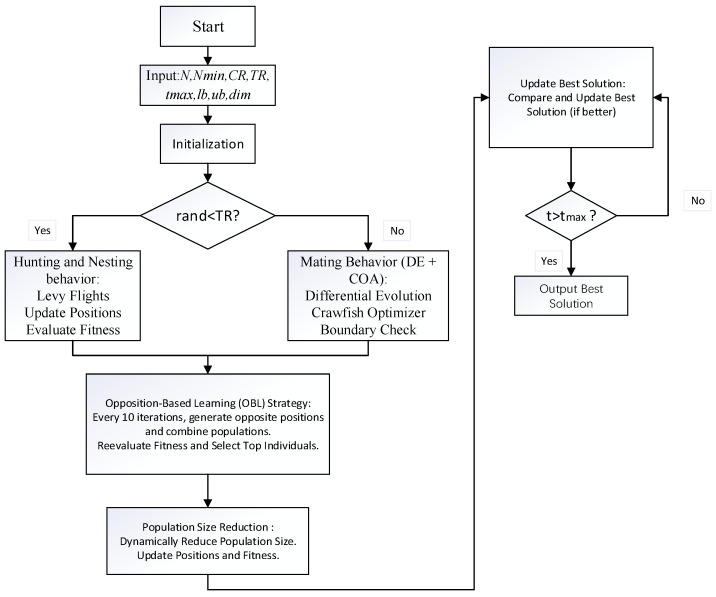
Flowchart of the ISWO algorithm.

The workflow of the improved algorithm is as follows. Initialization phase: The algorithm begins by generating an initial population of *n* individuals, each with a dimensionality of *d*, and positions randomly distributed within the defined upper and lower bounds. Parameters such as TR, Cr, and mutation factors are initialized. Through the opposition-based learning strategy, the population size is expanded by computing the opposite solutions for each individual. The original population is merged with these opposite solutions to form a new population. Fitness evaluations are performed on this new population, and the *n* individuals with the best fitness are selected as the initial population, initializing the optimal solution. Main loop phase: In each iteration of the generation, parameters are updated based on the current iteration count *t*. The dynamic adjustment of these parameters helps balance the trade-off between the exploration and exploitation phases. With a certain probability, the algorithm executes the exploration phase (hunting and nesting behaviors). During this phase, individuals update their positions according to Lévy flight patterns or based on the differences with other individuals. By calculating randomly generated parameters, the movement strategies of individuals are determined, aiming to increase population diversity and enhance global search capabilities. If the conditions for the exploration phase are not met, the exploitation phase is executed. At this stage, the algorithm integrates the characteristics of Differential Evolution (DE) and the Crayfish Optimization Algorithm (COA) to update individuals. After every 10 generations, the algorithm applies the opposition-based learning (OBL) strategy. At this point, opposite solutions for the individuals in the population are regenerated and combined with the current population to update the optimal solution. The OBL strategy aids in expanding the coverage of the search space, improving the algorithm’s diversity and global search ability. At the end of each generation, the algorithm adjusts the population size based on the current generation number *t*. Starting with an initial population size of *n*, the population size gradually decreases as the iterations progress until it reaches the minimum population size. This size adjustment strategy assists in conducting extensive exploration during the early stages and facilitates local exploitation in the later stages. Subsequently, the optimal solution of each generation is stored, and the optimal fitness value of the current generation is calculated to form a convergence curve. This curve reflects the algorithm’s convergence speed during the search process and the evolution of the optimal solution. When the maximum number of iterations $t_{max}$ is reached, the algorithm terminates. At this point, the optimal solution obtained is the final result of the algorithm. The specific algorithmic workflow is depicted in Figure 5.

### 3.1. An Enhancement Has Been Made to Equation (25)

In the original equation, N appears on both sides, which may lead to inaccurate calculations of N during each iteration since N is continuously updated throughout the iterative process. This recursive form can cause the population size to decrease either too rapidly or too slowly. To address this issue, the improved version of the original Equation (25) replaces the current N with the initial population size $N_{0}$. This modification ensures that the population size decreases linearly from the initial value to the minimum value as intended. The improved equation is as follows:

(26) $$N=N_{\min}+(N_{0}-N_{\min})\times \left(1-\frac{t}{t_{\max}}\right)$$

**Figure 6:**
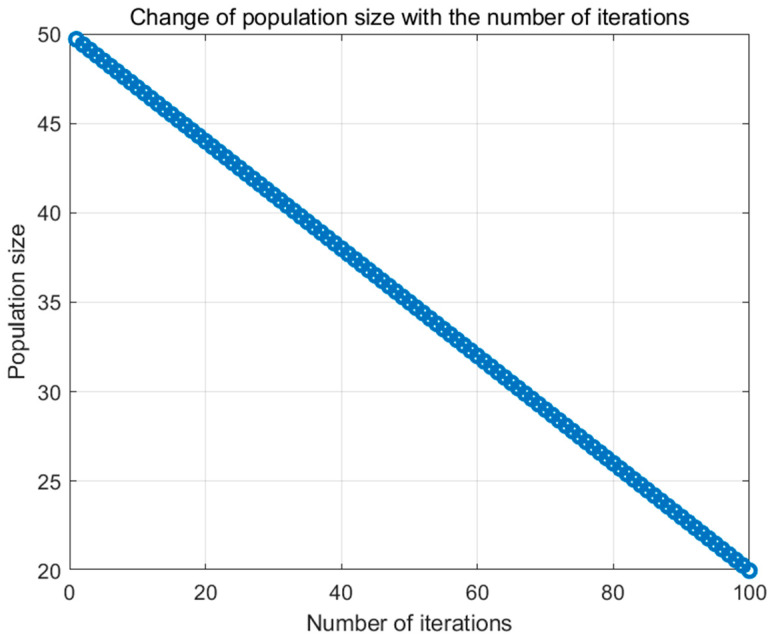
The improved Formula (25) accompanies the change in population size throughout the iterations of the ISWO algorithm.

Here, $N_{0}$ denotes the initial population size (i.e., the population size at t=0), and $t_{\max}$ signifies the maximum number of iterations. As illustrated in Figure 6, Equation (26) demonstrates that the population size progressively diminishes with increasing iterations. A larger population size during the initial stages facilitates extensive exploration of the solution space, thereby avoiding local optima. Conversely, a smaller population size in the later stages concentrates the search within promising regions, thereby accelerating convergence. This gradual reduction mechanism reduces computational overhead, automates the adjustment of population size, minimizes the complexity of manual parameter tuning, and effectively balances exploration and exploitation. Consequently, it enhances the overall performance of the algorithm and aids in locating the global optimal solution.

### 3.2. Adaptive Parameter Adjustment of TR and Cr

TR (trade-off probability) governs the balance between hunting and mating behaviors within the algorithm. Cr (crossover probability) controls the likelihood of crossover operations. As the number of iterations increases, gradually decreasing TR and Cr allows the algorithm to be more exploratory in the early stages and more exploitative in the later stages, thereby enhancing the convergence speed and solution accuracy. Consequently, a linear decay approach is employed, reducing these parameters incrementally from their initial values to zero. Here, t represents the current iteration count, and $t_{\max}$ denotes the maximum number of iterations.

(27) $$\mathrm{TR}=\mathrm{TR}\left(0\right)\times \left(1-\frac{t}{t_{\max}}\right)$$

In Equation (27), TR(0) represents the initial behavioral weight, $t_{\max}$ denotes the maximum number of iterations, and t refers to the current iteration.

(28) $$\mathrm{Cr}=\mathrm{Cr}\left(0\right)\times \left(1-\frac{t}{t_{\max}}\right)$$

In Equation (28), Cr(0) represents the initial crossover rate, $t_{\max}$ denotes the maximum number of iterations, and t refers to the current iteration.

**Figure 7:**
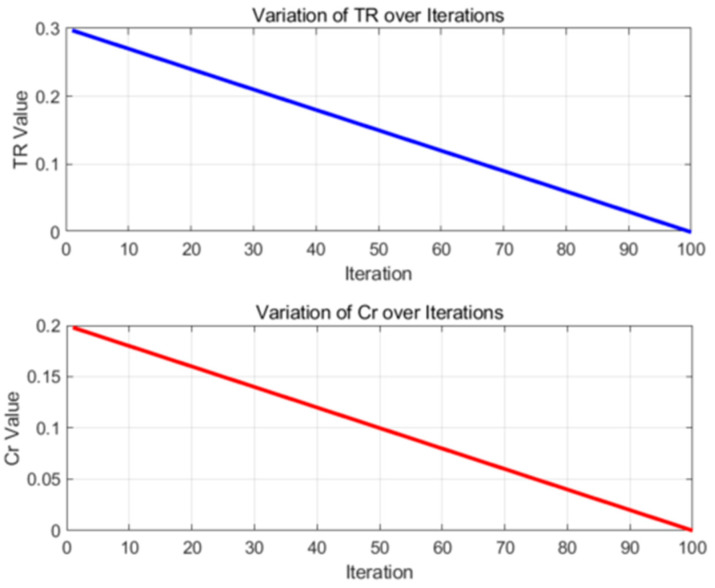
The adaptive adjustments of the TR and Cr parameters over 100 iterations illustrate their variations throughout the iterative process.

As illustrated in Figure 7, within the various stages of the Improved Spider Wasp Optimizer (ISWO), the parameters TR and Cr are dynamically adjusted in response to the number of iterations to balance exploration and exploitation.

Initial phase (first 20 iterations): TR begins at 0.3 and gradually decreases, while Cr starts at 0.2 and similarly decreases. During this phase, the algorithm predominantly engages in hunting and nesting behaviors, as well as crossover operations. This inclination enhances the diversity of solutions and facilitates an extensive search of the solution space.

Intermediate phase (around the 50th iteration): Both TR and Cr are reduced by approximately half. The algorithm begins to balance exploration and exploitation, continuing to search new regions while also initiating fine-tuning of existing solutions.

Final phase (last 20 iterations): TR and Cr approach zero. At this stage, the algorithm primarily performs mating behaviors with a lower probability of crossover operations, concentrating on the meticulous refinement of the current optimal solution to enhance its precision.

The role of TR here is to control the probability of the algorithm selecting between hunting and nesting behaviors versus mating behaviors. A high TR value biases the algorithm towards global exploration, which aids in escaping local optima. Conversely, a low TR value favors local exploitation, accelerating the convergence speed.

Therefore, the adaptive adjustment of TR enables the algorithm to conduct a broad search of the solution space during the initial phase, preventing premature convergence. In the later stages, it shifts towards the fine-tuning of the current solution, thereby increasing the precision of the solution and speeding up convergence.

### 3.3. Integration of the Crayfish Optimization Algorithm

The Crayfish Optimization Algorithm (COA) simulates the foraging and avoidance behaviors of crayfish, utilizing both local and global search strategies to further optimize the positions of individuals in the original SWO algorithm, improving search efficiency and accuracy. When executing the ISWO algorithm, the first step is to determine whether to perform COA behaviors. During each iteration of the main loop, the algorithm initially decides whether to execute the “hunting and nesting behaviors” based on a randomly generated probability rand within the range [0, 1], specifically if rand<TR. If the condition rand<TR is not met, the algorithm proceeds to the mating behavior phase. In the mating phase (when COA behavior is selected), a random value temp is generated using a specified formula to decide whether to execute specific COA behaviors. If temp>30, the COA update formulas are employed: using COA’s Equations (32) and (34), the positions of the spider wasps are updated. Here, $C_{2}$ is a control coefficient that gradually decreases as the number of iterations increases. The updated position $\vec{{SW}_{m}}$ is generated through a linear combination with the optimal solution $\vec{{SW}^{*}}$. If temp ≤ 30, a “fitness parameter” p is calculated based on the current value. The update position is then generated using $X_{food}$, which is an update formula based on p. This formula adjusts the fitness based on the objective function values to generate new food positions. Ultimately, $\vec{{SW}_{m}}$ is updated as a linear combination based on the optimal solution $\vec{{SW}^{*}}$ and the current solution. Below are the referenced COA formulas.

In Equation (29), temperature changes influence the foraging behavior of crayfish, thereby controlling the balance between exploration and exploitation. Here, temp represents the temperature of the crayfish’s environment.

(29) temp=rand×15+20

Here, rand denotes a random number, and temp is a value generated from a uniformly distributed random number within the range [20, 35].

In the Improved Spider Wasp Optimizer (ISWO) algorithm, the generation of temp directly influences the position-update rules of the search agents. When temp is high, the system may adopt more aggressive exploitation behaviors (i.e., conducting local searches near the optimal solution). Conversely, a lower temperature implies that the system will engage in broader exploratory behaviors.

**Figure 8:**
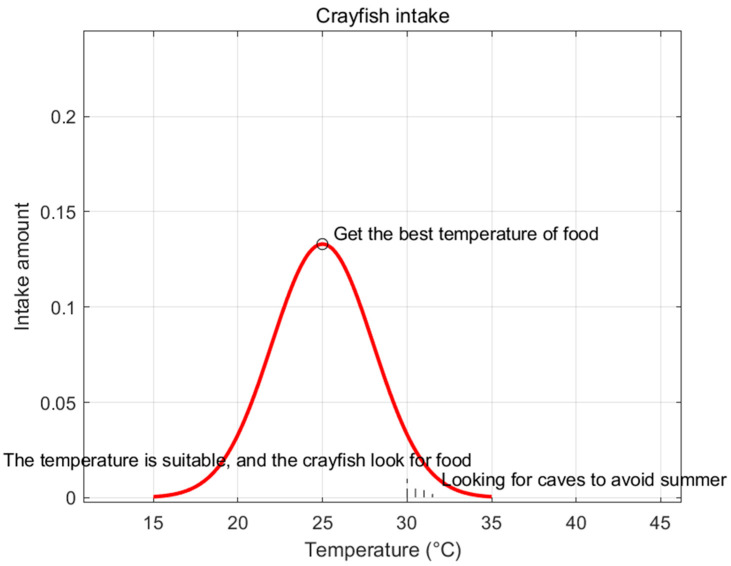
Effect of temperature on crayfish intake.

The mathematical model of crayfish foraging is presented in Equation (30). An illustrative diagram of food intake is shown in Figure 8.

(30) $$p=C_{1}\times (\frac{1}{\sqrt{2\times \pi }\times \sigma )}\times \exp(-\frac{{\left(temp-\mu \right)}^{2}}{2\sigma^{2}})).$$

Here, μ represents the optimal temperature for crayfish, which corresponds to their fitness. The parameters σ and $C_{1}$ are utilized to control the crayfish’s foraging rate at different temperatures, thereby influencing the fitness of the optimal solution under varying temp conditions. The value of p is calculated based on the fitness of the current search agent $\vec{{SW}_{m}}$ and the fitness of the global optimal solution $\vec{{SW}^{*}}$. The p value is employed to determine whether to proceed with more intensive exploratory behaviors (such as executing larger jumps or focusing on the local region of the current solution). A larger p value tends to encourage more exploratory behaviors, while a smaller p value promotes more exploitative behaviors.

(31) $$X_{shade}=\left(\begin{array}{c} X_{G}+X_{L} \end{array}\right)/2,$$

In Equation (31), $X_{shade}$ serves as the midpoint between the current individual’s position $X_{L}$ and the global optimal solution $X_{G}$. It represents the direction in which the current individual moves toward the global optimum. By calculating the midpoint of $X_{G}$ (the global optimal solution) and the current position $X_{L}$, $X_{shade}$ is used as a reference point for updating positions. This indicates that the Crayfish Optimization Algorithm leverages global information to guide individuals toward the optimal solution.

(32) $$X_{i,j}^{t+1}=X_{i,j}^{t}+C_{2}\times rand\times \left(X_{shade}-X_{i,j}^{t}\right)$$

In Equation (32) of the ISWO algorithm, $\vec{{SW}_{m}}$ represents the updated individual position $X_{i,j}^{t+1}$. This update is achieved by calculating the distance between the individual’s current position $X_{i,j}^{t}$ and the midpoint $X_{shade}$ of the global optimal solution, employing a scaling factor $C_{2}$. Equation (34) embodies the global optimization mechanism during the exploration process. The parameter $C_{2}$ controls the search step size, and the random number rand is used to introduce variations across different dimensions. This enhances the algorithm’s diversity, enabling rapid jumps on a global scale to explore potential global optimal solutions.

(33) $$C_{2}=2-(t/t_{\max})$$

In Equation (33), $C_{2}$ is a scaling factor that varies with the number of iterations and gradually decreases as the iterative process progresses. $C_{2}$ adjusts the magnitude of the individuals’ jumps within the search space. During the initial phase of the algorithm, a larger $C_{2}$ implies that individuals have larger step sizes, allowing them to explore a more expansive region. As time advances, $C_{2}$ diminishes, indicating that the search process is gradually converging, and individuals conduct fine-grained searches within smaller areas. This helps balance the relationship between global exploration and local exploitation. Here, $t_{max}$ denotes the maximum number of iterations.

(34) $$X_{i,j}^{t+1}=X_{i,j}^{t}-X_{z,j}^{t}+X_{shade}$$

In Equation (34), the position is updated by calculating the difference between the current individual position $\vec{{SW}_{m}}$ and the position $X_{z,j}^{t}$ of another randomly selected individual, then adding $X_{shade}$. This update method enables the individual to rely not only on the global optimal solution $\vec{{SW}^{*}}$ but also incorporates the influence of neighboring individuals (through the random selection of z). This approach enhances the diversity of the search, rendering the individual’s exploratory behavior more flexible and preventing premature convergence.

(35) $$X_{food}=\exp\left(-\frac{1}{p}\right)\times X_{food}$$

In Equation (35), the right-hand-side term $X_{food}$ represents the global optimal solution $\vec{{SW}^{*}}$ in the ISWO algorithm. This equation encapsulates how the crayfish locates a highly attractive food source during its search process. Based on the value of p, the expression exp(−1 / p) reduces the distance to the global optimal solution $\vec{{SW}^{*}}$, thereby attracting individuals toward it. When p is large, individuals exhibit a broader search scope, indicating global exploration; when p is small, it signifies more intensive local exploitation.

(36) $$X_{i,j}^{t+1}=X_{i,j}^{t}+X_{food}\times p\times (\cos(2\times \pi \times rand)-\sin(2\times \pi \times rand))$$

In Equation (36), $\vec{{SW}_{m}}$ in the ISWO algorithm represents the updated individual position $X_{i,j}^{t+1}$. $X_{food}$ is the global optimal solution $\vec{{SW}^{*}}$ at generation t. The position $X_{i,j}^{t+1}$ adjusts based on the attraction of the global optimal solution $X_{food}$ and incorporates a random term weighted by p.

(37) $$X_{i,j}^{t+1}=\left(\begin{array}{c} X_{i,j}^{t}-X_{food} \end{array}\right)\times p+p\times rand\times X_{i,j}^{t}$$

In Equation (37), $X_{i,j}^{t+1}$ is updated by amalgamating the positional difference between the global optimal solution and the current individual. This equation embodies the hybrid strategy in the Crayfish Optimization Algorithm (COA), wherein the global optimal solution $\vec{{SW}^{*}}$ directs the search while a stochastic term enhances search diversity. Here, p serves as a scaling factor, controlling the intensity of the current search.

### 3.4. Integration of Differential Evolution Algorithm

Differential Evolution (DE) generates new candidate solutions through mutation and crossover operations, utilizing the differential information between individuals in the population, which enhances the population diversity and search capability compared to the original SWO algorithm. The synergy between COA and DE accelerates the discovery of the optimal solution and the convergence process. The introduction of DE enhances the exploration capability of the ISWO algorithm and, when combined with COA, further improves the algorithm’s performance and convergence speed.

The evolutionary process of the DE algorithm in ISWO is as follows.

First, the mutation operation is performed, where a new individual is generated by computing the difference between three randomly selected individuals from the population. The specific mutation formula is as follows:

(38) $$V=X_{r1}+F\cdot (X_{r2}-X_{r3})$$

In Equation (38), V represents the mutant individual. $X_{r1},X_{r2},X_{r3}$ are three different individuals randomly selected from the population. F is the mutation factor that controls the magnitude of the mutation.

Next, a crossover operation is performed on each dimension of the mutant individual V and the current individual X. If a random number is less than the crossover probability Cr, the value is taken from the mutant individual V; otherwise, the value from the current individual X is retained. The specific formula is as follows:

(39) $$U_{j}=\left\{\begin{array}{c} V_{j}\;\mathrm{if}\;rand<\mathrm{Cr}\\ X_{j}\;\mathrm{otherwise} \end{array}\right.$$

In Equation (39), $U_{j}$ represents the candidate solution after the crossover operation. Cr is the crossover probability. $V_{j}$ is the j-th dimension of the mutant individual, and $X_{j}$ is the j-th dimension of the current solution. rand is a uniformly distributed random number within the interval [0, 1].

Finally, a selection operation is performed to decide whether to update the current position. If $f\left(U\right)<f(X)$, the candidate solution U is selected; if $f\left(U\right)>f(X)$, the candidate solution X is chosen. Here, $f\left(U\right)$ and f(X) denote the fitness values.

The update results from the DE algorithm and the COA algorithm are integrated as shown in Equation (40).

(40) $$X_{i,j}^{i}=(X_{i,j}^{i}+\vec{SW_{m}})/2$$

In the expression $\left(X_{i,j}^{i}+\vec{{SW}_{m}}\right)$, $X_{i,j}^{i}$ represents the current position of individual i (i.e., the solution vector), and $\vec{{SW}_{m}}$ denotes the new solution obtained after simulating crayfish behavior based on COA. The term $(X_{i,j}^{i}+\vec{{SW}_{m}})/2$ signifies an averaging operation that combines the results from both the DE and COA components. Specifically, the solution provided by the DE part is derived from global search capabilities, while the COA part offers a solution based on local search and the simulated foraging behavior of crayfish. The DE contributes by enhancing the global search ability, enabling individuals to escape local optima. In contrast, the COA’s contribution focuses on meticulous local search, allowing the solution to be refined near the optimal region.

### 3.5. Perform Dynamic Readjustment of the Adaptive Parameters TR and Cr

In the ISWO algorithm, dynamic updates of TR and Cr are implemented to optimize the algorithm’s performance during the search process by adjusting exploration and exploitation behaviors. Specifically, this dynamic update design is based on the following two considerations: First, adjusting the balance between exploration and exploitation. In optimization algorithms, exploration and exploitation are two critical processes. In the early stages, the algorithm requires more exploration to avoid premature convergence to local optima; in the later stages, it should focus more on exploitation to refine the current optimal solution and ultimately converge to the global optimum.

By dynamically adjusting TR and Cr, the algorithm’s search strategy can gradually change during different iterative stages. TR decreases linearly from ${\mathrm{TR}}_{\max}$ to ${\mathrm{TR}}_{\min}$; this means that over time, the algorithm gradually reduces the proportion of hunting and nesting behaviors while increasing the proportion of mating behaviors. This allows the algorithm to explore the search space more extensively in the initial stages to avoid falling into local optima, and to focus on fine-tuning the current optimal solution in the later stages. Cr increases linearly from ${\mathrm{Cr}}_{\min}$ to ${\mathrm{Cr}}_{\max}$: As the iterations progress, the algorithm gradually increases the frequency of crossover operations. This helps maintain diversity during the early exploration phase, preventing premature convergence; in the later stages, stronger crossover operations promote solution fusion and optimization, accelerating the convergence speed.

Second, emphasizing exploration in the early stages and exploitation in the later stages. The dynamic updating strategy of TR and Cr is based on the different requirements of the algorithm behaviors at various stages. Early stage ($t<t_{\max}$ with smaller t values): At this stage, search agents rely more on exploration behaviors. By employing a higher TR (favoring hunting and nesting behaviors) and a lower Cr (less crossover), the algorithm can effectively avoid becoming trapped in local optima and extensively explore the solution space. Later stage (t approaching $t_{\max}$): Over time, the algorithm gradually shifts towards more exploitation behaviors, increasing Cr to enhance the refinement of solutions, and decreasing TR to focus on local optimization and convergence.

Therefore, the original Equations (27) and (28) need to be improved to Equations (41) and (42).

(41) $$\mathrm{TR}={\mathrm{TR}}_{\min}+({\mathrm{TR}}_{\max}-{\mathrm{TR}}_{\min})\times \left(1-\frac{t}{t_{\max}}\right)$$

(42) $$\mathrm{Cr}={\mathrm{Cr}}_{\min}+({\mathrm{Cr}}_{\max}-{\mathrm{Cr}}_{\min})\times \left(\frac{t}{T_{\max}}\right)$$

**Figure 9:**
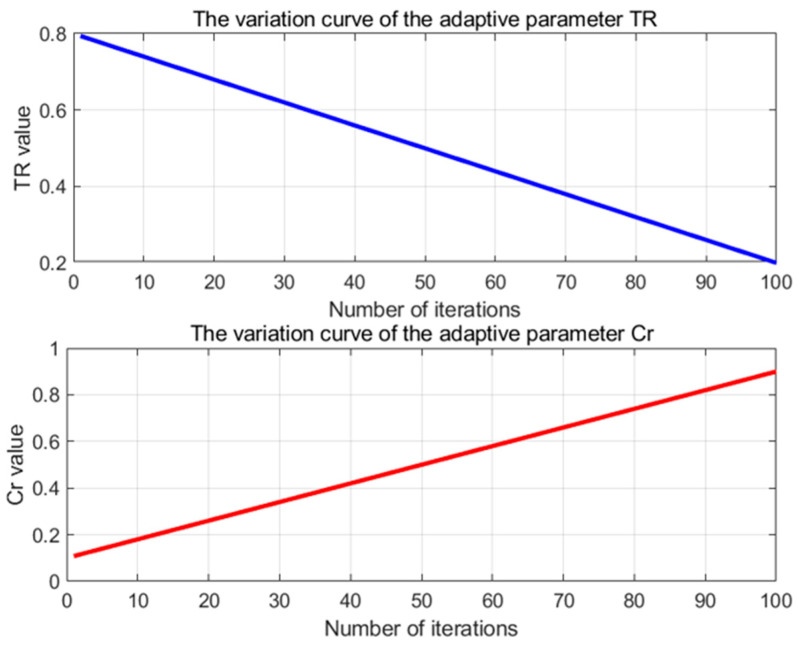
The adaptive adjustments of the TR and Cr parameters over 100 iterations illustrate their variation throughout the iterative process.

Figure 9 depicts the variations in the adaptively adjusted TR and Cr parameters over 100 iterations after their readjustment. The TR parameter decreases linearly from 0.8 to 0.2, which implies that in the early stages, the algorithm tends to perform more exploratory behaviors (hunting and nesting) to extensively search the solution space and avoid becoming trapped in local optima. As the iterations advance, the TR value gradually diminishes, making the algorithm more inclined to execute exploitative behaviors in the later stages, focusing on refining the current optimal solution to achieve better convergence. This strategy ensures diversity during the initial optimization phases while emphasizing convergence in the later stages.

Similarly, the Cr parameter increases linearly from 0.1 to 0.9, indicating that as the iterations progress, the algorithm gradually increases the probability of crossover operations. In the early stages, a smaller Cr maintains solution diversity, preventing premature convergence. In the later stages, the increase in Cr allows solutions to merge more effectively through crossover during local exploitation, thereby accelerating convergence and improving optimization precision.

### 3.6. Incorporation of the Centroid Opposition-Based Learning Strategy

The opposition-based learning (OBL) strategy expands the search space by generating the “opposite” solution of the current solution, which helps the improved SWO algorithm escape local optima and enhances its global search capability. The core idea of this formula is to produce an opposite solution based on the current solution and the problem’s lower and upper bounds (lb and ub), thereby enhancing population diversity and preventing the algorithm from becoming trapped in local optima.

(43) $$x_{i}^{\prime }=lb_{i}+ub_{i}-x_{i}$$

In Equation (43), $x_{i}$ is the value of the current solution at the i-th dimension. ${lb}_{i}$ and ${ub}_{i}$ are the lower and upper bounds of the i-th dimension, respectively. $x_{i}^{\prime }$ is the opposite solution of the current solution x at the i-th dimension.

**Figure 10:**
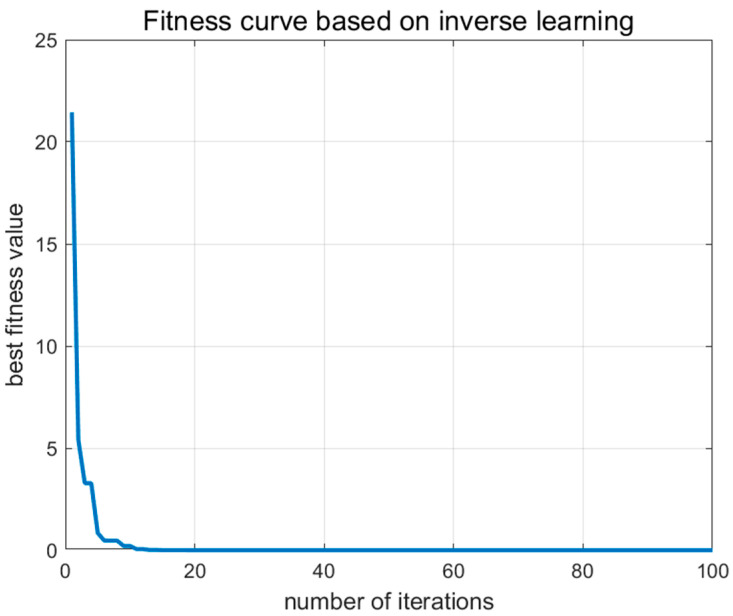
Fitness curve based on inverse learning.

According to Figure 10, the impact of the centroid opposition-based learning (OBL) strategy within the ISWO algorithm is primarily manifested through the incorporation of opposite solutions, which accelerates the algorithm’s convergence toward near-optimal solutions in its early stages. Opposition-based learning enhances the coverage of the solution space, thereby increasing population diversity and preventing the algorithm from prematurely converging to local optima. The application of OBL throughout the iterative process ensures the smoothness and stability of the algorithm’s convergence (Algorithm 2).
**Algorithm 2** The Improved Spider Wasp Optimization AlgorithmInput:$N,\;Nmin,\;\mathrm{CR},\;\mathrm{TR},\;t_{max},lb,ub,dim,$Output:$\vec{{SW}^{*}}$1.Initialize parameters2. Set boundary conditions lb,ub3. Initialize $\vec{{SW}^{*}}$ as a zero vector4. Set optimal fitness to infinity5.Initialize positions of search agents6. $\mathrm{Use}\;\mathrm{initialization}\;\mathrm{function}\;\mathrm{to}\;\mathrm{generate}\;\mathrm{initial}\;\mathrm{positions}\;x_{i}$
7. $\mathrm{Generate}\;\mathrm{an}\;\mathrm{opposite}\;\mathrm{population}\;\mathrm{using}\;\mathrm{opposition}\;\mathrm{learning}\;x_{i}^{\prime }$
8. Combining the original and inverse populations9. $\mathrm{Evaluate}\;\mathrm{each}\;\vec{{SW}_{i}}$ $\mathrm{and}\;\mathrm{finding}\;\mathrm{the}\;\mathrm{one}\;\mathrm{with}\;\mathrm{the}\;\mathrm{best}\;\mathrm{fitness}\;\mathrm{in}\;\vec{{SW}^{*}}$
10.Main loop (t=0 $\mathrm{to}\;t_{max}$)11. To calculate the values of TR, Cr, and k using Equations (26), (41) and (42),12. Randomly shuffle the index13. Judgement of hunting and nesting behavior based on TR14.  rand between 0 and 115.  If rand<TR, perform hunting and nesting behavior16.  $\mathrm{The}\;\mathrm{optimal}\;\mathrm{solution}\;\vec{{SW}^{*}}$ can be calculated using Formulas (4)–(8) from the exploration phase and Formulas (10), (12), (16) and (17) from the follow-escape phase.17.  Perform boundary check18.  Else, perform mating behavior (DE + COA hybrid strategy) 19.  Differential Evolution (DE) operates using Equations (38) and (39)20.  Perform crossover operations21.  Apply boundary check22.  $\mathrm{The}\;\mathrm{optimal}\;\vec{{SW}^{*}}$ solution can be obtained by combining COA using Formulas (29)–(37), and then fusing the results of DE and COA, applying Formula (40).23.Apply opposition-based learning (OBL) every 10 iterations24. $\mathrm{Generate}\;\mathrm{an}\;\mathrm{opposite}\;\mathrm{population}\;\mathrm{using}\;\mathrm{opposition}\;\mathrm{learning}\;x_{i}^{\prime }$
25. Combining the original and inverse populations26. if $f\left(U\right)<f(X)$27.  $X=\vec{{SW}^{*}}$
28. Else, *U* $=\vec{{SW}^{*}}$29.Reduce population size30. Compute the new population size ensuring it is not less than the minimum size31. Update the population accordingly

## 4. Simulation Tests and Result Analysis of the Improved Spider Wasp Optimizer Algorithm

To validate the performance of the Improved Spider Wasp Optimizer (ISWO), it was compared with five other popular algorithms introduced in the past two years: the Crayfish Optimization Algorithm (COA), the Spider Wasp Optimizer (SWO), the Black-winged Kite Algorithm (BKA), the Catch Fish Optimization Algorithm (CFOA), and the Hippopotamus Optimization Algorithm (HO). The following sections describe the test functions used for the experimental data, the comparative algorithms, parameter configurations, and the analysis of the experimental results. This experiment was conducted on a Thunderobot laptop in Deyang, China. The computer was equipped with a 12th Gen Intel(R) Core i5-12450H processor (base frequency 2.50 GHz) and 256 GB of memory, running the MATLAB R2023b software.

### 4.1. Introduction to the CEC2017 Test Suite and Algorithm Parameters

The CEC2017 benchmark test suite comprises 30 optimization test functions, widely used for evaluating and comparing the performance of optimization algorithms. These functions are categorized into four main classes based on their characteristics. F1–F3 unimodal functions: These functions have only one global optimum and are primarily used to test an algorithm’s exploitation capability and convergence speed. F4–F10 simple multimodal functions: Featuring multiple local optima, these functions assess an algorithm’s ability to avoid becoming trapped in local optima. F11–F20 hybrid functions: By combining different types of functions, these hybrid functions increase problem complexity, testing an algorithm’s capability to handle complex search spaces. F21–F30 composition functions: Composed of multiple basic functions with complex landscapes and diverse features, they are used for comprehensively evaluating an algorithm’s global search and local exploitation abilities. The numbers in the last column represent the benchmark optimal values Fi* for the CEC’17 test functions, as shown in Table 1. These values indicate the function’s value, *Fi*(x*), at the global optimal solution x*.

In this study, the parameters of each algorithm in the CEC2017 test suite are provided in Table 2. In Table 2, the CFOA parameter Efs represents the number of objective function evaluations.

### 4.2. Analysis of the Test Results for Each Algorithm

Firstly, from the data of the 50-dimensional tests in Table A1, it is evident that the minimum values (Min) and mean values (Mean) are lower for the ISWO algorithm. In most test functions (F1–F30), the ISWO algorithm consistently achieves lower minimum and mean values compared to other algorithms. This indicates that the ISWO algorithm excels in finding global optimal solutions, yielding superior results. Additionally, the standard deviation of the ISWO algorithm is typically lower, signifying reduced variability in its outcomes and demonstrating more stable performance.

**Figure A1:**
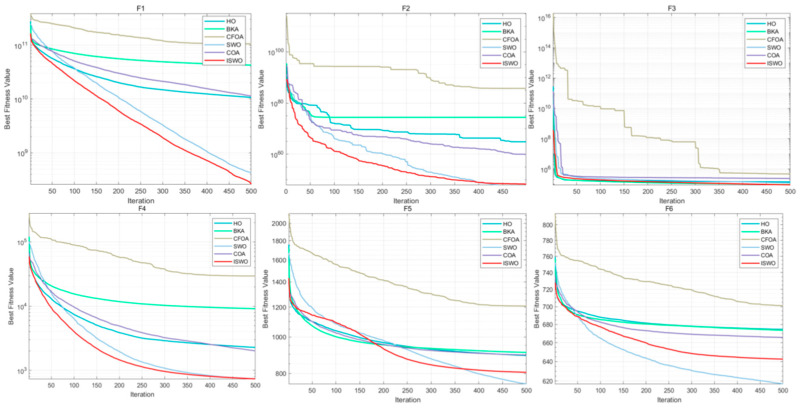
F1–F6 (50 dims).

**Figure A2:**
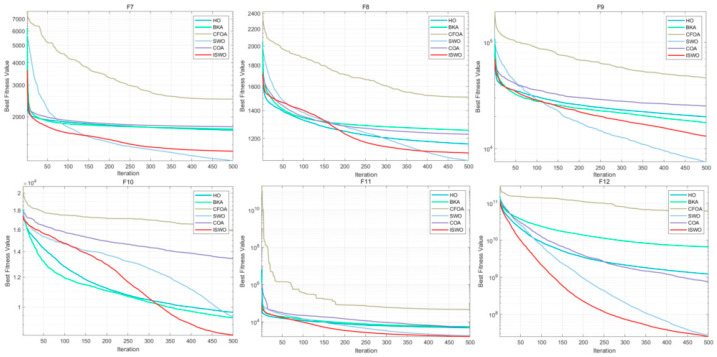
F7–F12 (50 dims).

**Figure A3:**
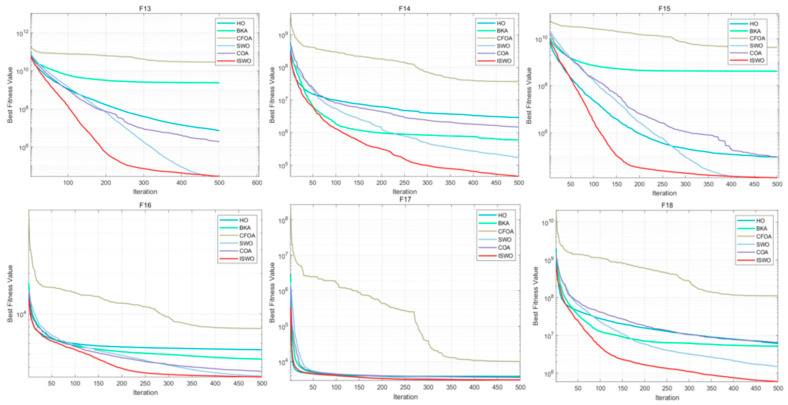
F13–F18 (50 dims).

**Figure A4:**
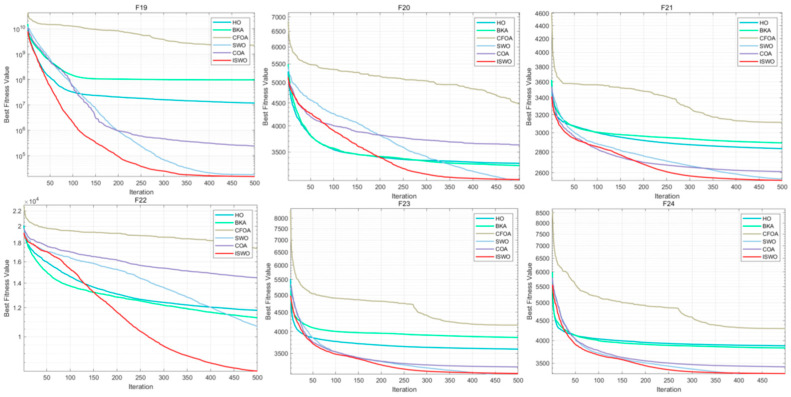
F19–F24 (50 dims).

**Figure A5:**
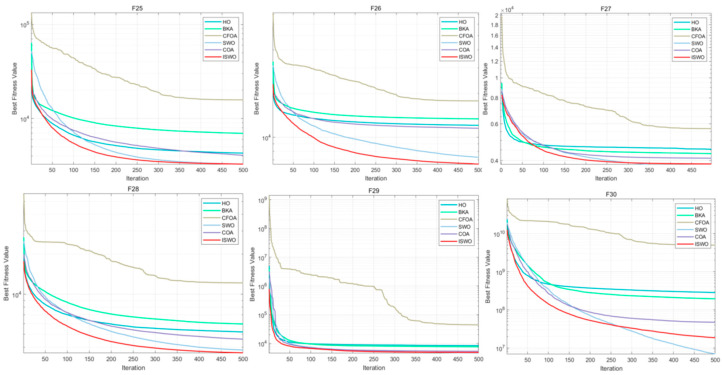
F25–F30 (50 dims).

**Figure A6:**
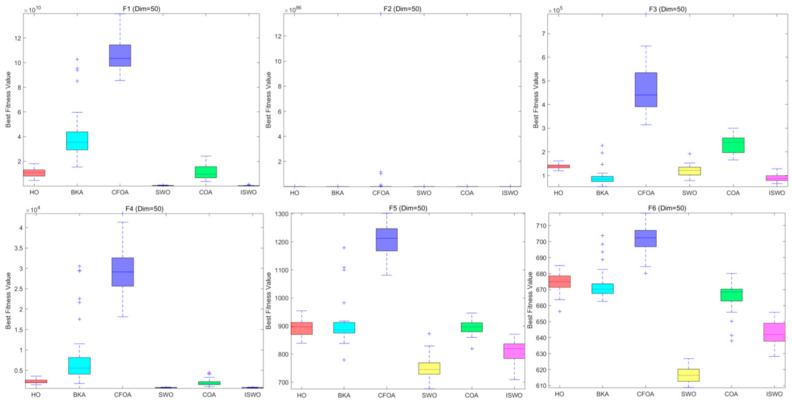
F1–F6 (50 dims) box plot (math.).

**Figure A7:**
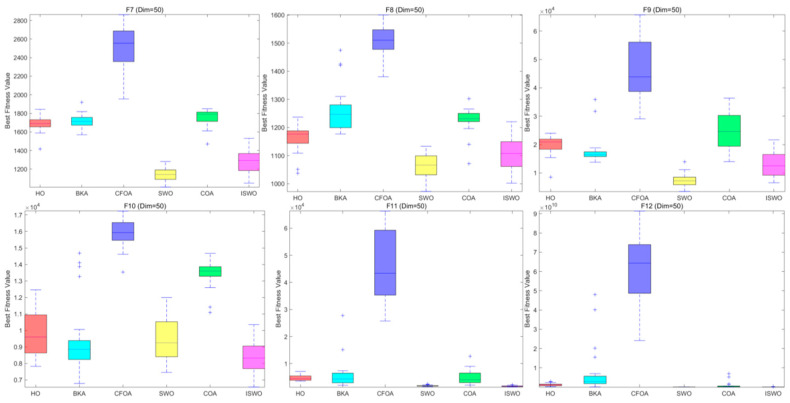
F7–F12 (50 dims) box plot (math.).

**Figure A8:**
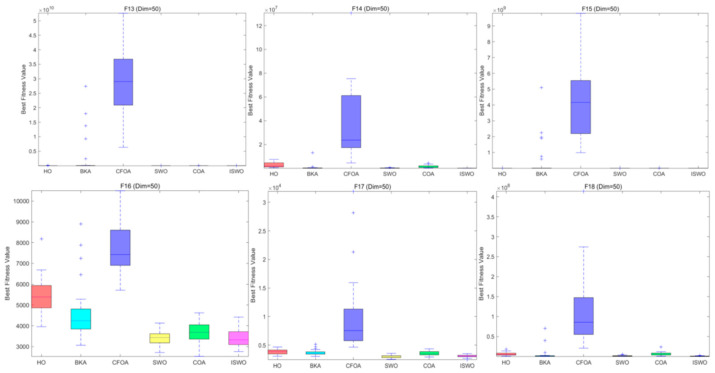
F13–F18 (50 dims) box plot (math.).

**Figure A9:**
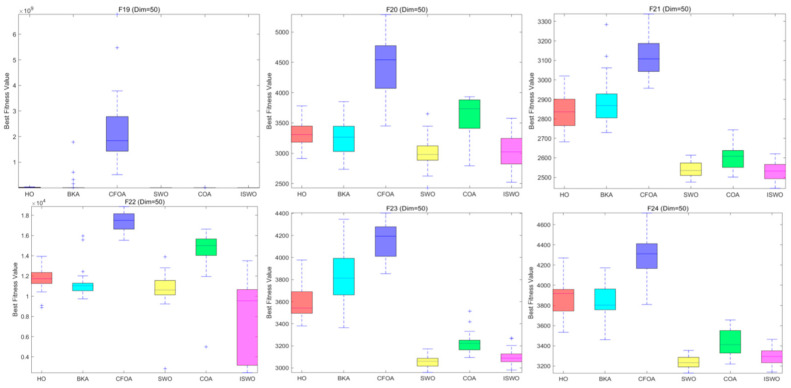
F19–F24 (50 dims) box plot (math.).

**Figure A10:**
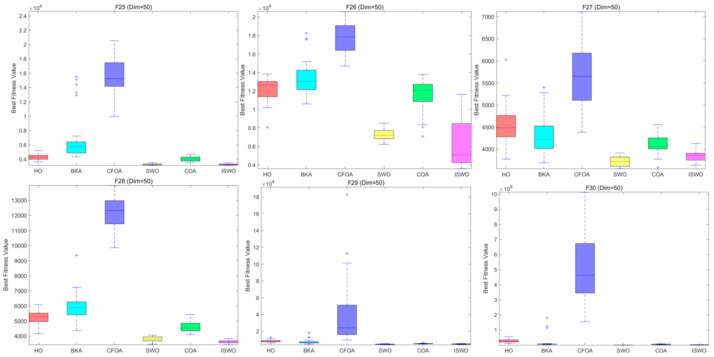
F25–F30 (50 dims) box plot (math.).

The HO, BKA, and CFOA algorithms exhibit higher maximum, mean, and minimum values across multiple test functions, suggesting a tendency to become trapped in local optima and a lack of robust global search capability. Moreover, as observed from the convergence curves (Figure A1, Figure A2, Figure A3, Figure A4 and Figure A5) in Appendix C and the box plots (Figure A6, Figure A7, Figure A8, Figure A9 and Figure A10) in Appendix D, there is a significant disparity in iteration stability and the optimal values found between the ISWO algorithm and the HO, BKA, and CFOA algorithms. This is also reflected in the *p*-value analysis.

In the box plots of Figure A6 and Figure A7, the ISWO algorithm displays relatively shorter box lengths, indicating lower variance in the results and demonstrating stability, with better robustness. For instance, in functions F5, F8, and F11, the results of ISWO are notably concentrated, implying minimal performance fluctuations across multiple runs. Conversely, algorithms like BKA and CFOA show larger box lengths and more outliers in the box plots of several test functions (such as F1, F2, F3, and F8), which means their results are less stable and more susceptible to the influence of initial solutions, leading to significant performance variability and unstable optimization quality.

From Figure A5 and Figure A10 concerning function F30, it is apparent that the ISWO algorithm exhibits some shortcomings in the optimization and iteration processes. This is primarily because functions like F30 are high-complexity multimodal functions with numerous local extrema in their objective functions. The exploration process of ISWO tends to become trapped in these local optima, which imposes higher demands on the precision of the search. The global optimum of such functions is unevenly distributed, with many local optima that are relatively concentrated. Consequently, ISWO may spend extended periods near a local optimum, affecting its efficiency in swiftly locating the global optimum.

In the test curves for functions F5 and F6 in Figure A1, it can be seen that although ISWO performs relatively well overall, its convergence speed and final optimal value do not significantly surpass those of other algorithms. In F5, the descent curve of ISWO is similar to those of HO and SWO, indicating that it has not achieved a substantial advantage during the exploration process. The optimization of F5 and F6 may rely on the fine exploitation of local optima; such functions require the algorithm to continuously make precise adjustments during the search to find better solutions.

From Table A2 (100-dimensional) in Appendix B, it is evident that as the dimensionality increases, the complexity of the problem escalates, highlighting the strong adaptability of the ISWO algorithm in high-dimensional optimization problems. The *p*-value analysis shows that a *p*-value lower than 0.05 indicates a statistically significant difference, confirming that one algorithm outperforms another. Conversely, a *p*-value higher than 0.05 suggests no significant difference, and the observed variations may be attributed to randomness. In most functions, the ISWO algorithm exhibits a clear advantage.

**Figure A11:**
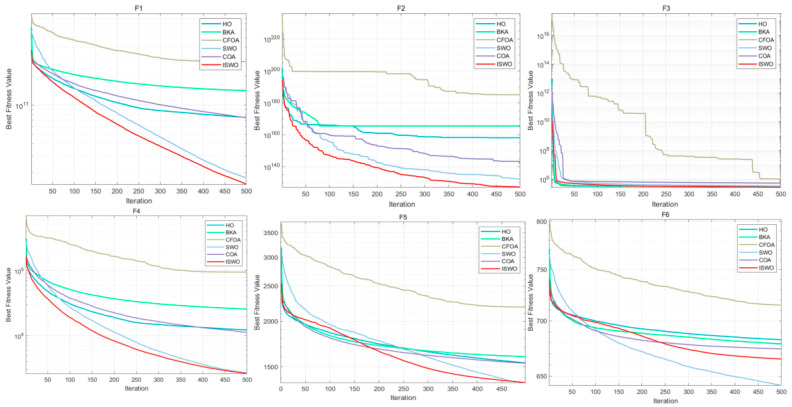
F1–F6 (100 dims).

**Figure A12:**
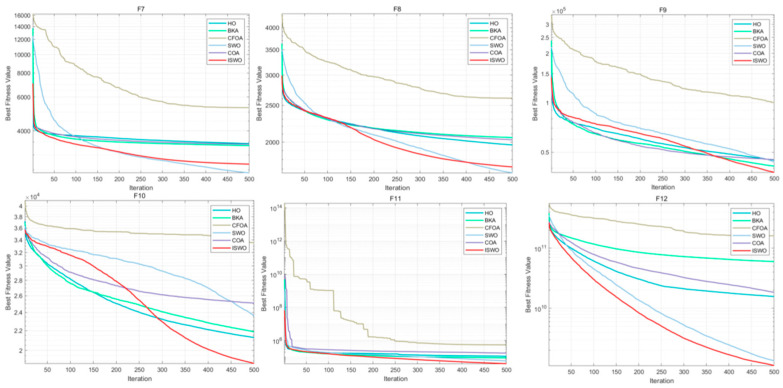
F7–F12 (100 dims).

**Figure A13:**
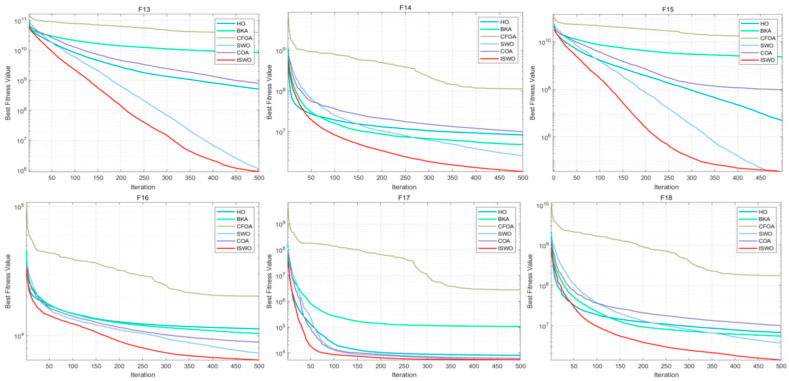
F13–F18 (100 dims).

**Figure A14:**
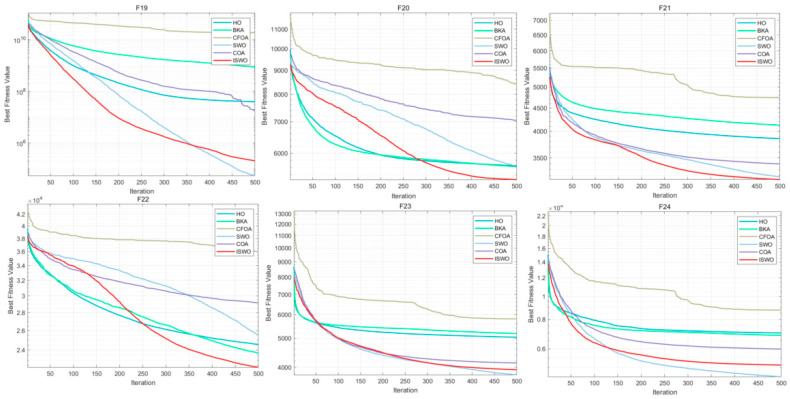
F19–F24 (100 dims).

**Figure A15:**
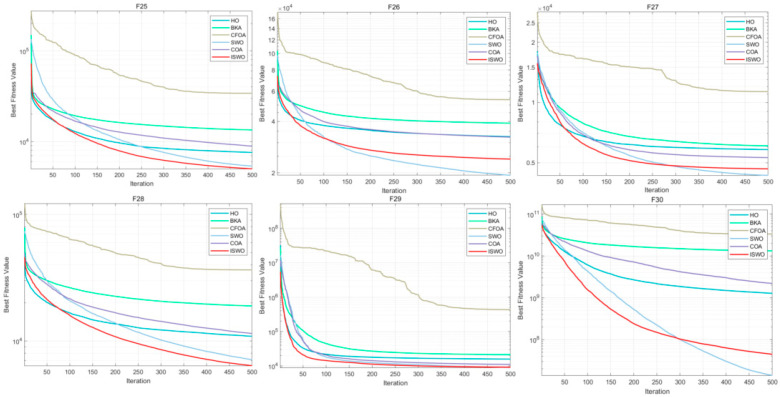
F25–F30 (100 dims).

**Figure A16:**
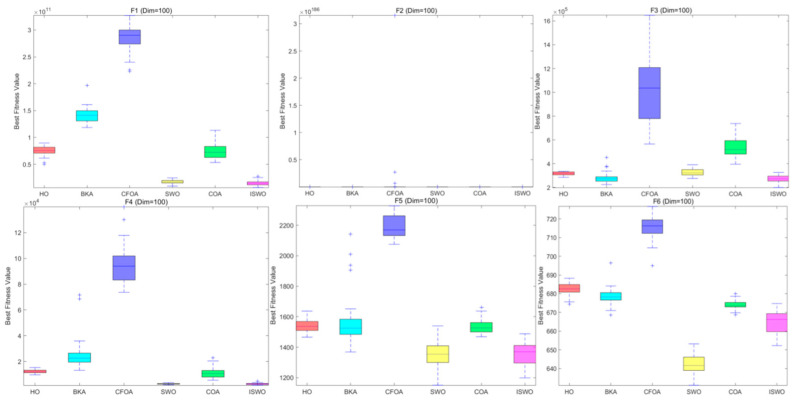
F1–F6 (100 dims) box plot (math.).

**Figure A17:**
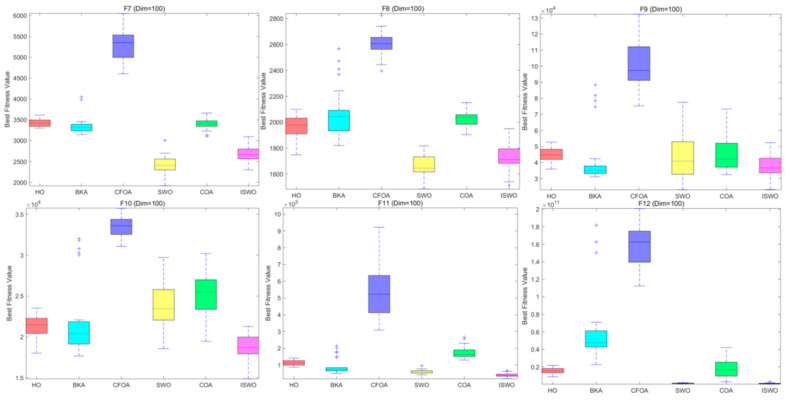
F7–F12 (100 dims) box plot (math.).

**Figure A18:**
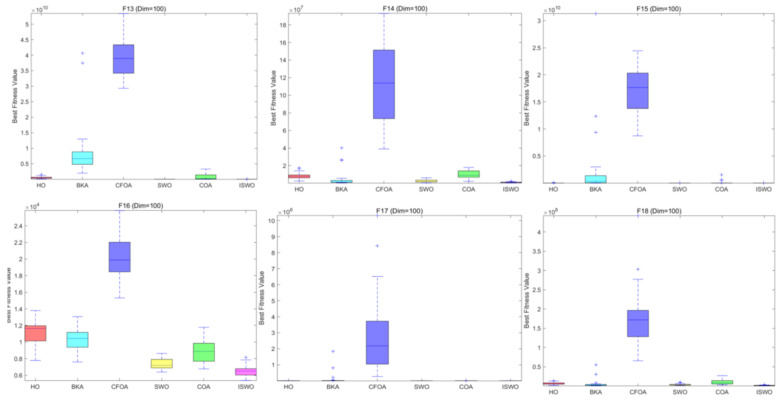
F13–F18 (100 dims) box plot (math.).

**Figure A19:**
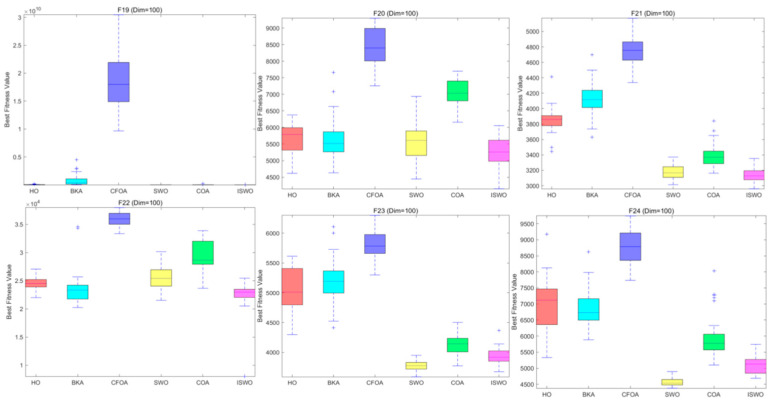
F19–F24 (100 dims) box plot (math.).

**Figure A20:**
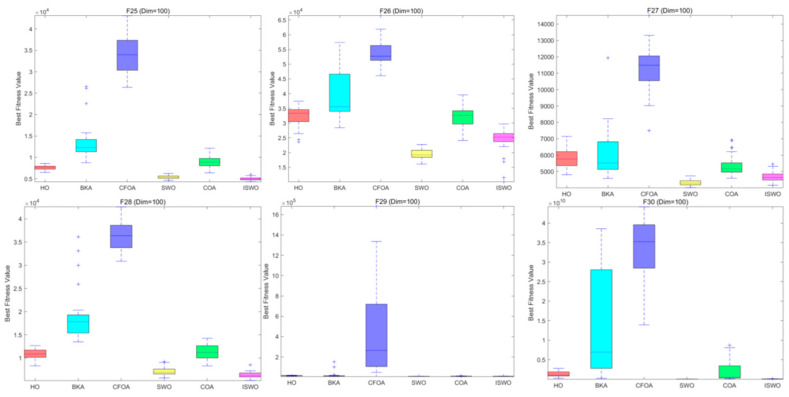
F25–F30 (100 dims) box plot (math.).

The standard deviation (Std) of the ISWO algorithm is generally small, indicating consistent results across multiple runs and demonstrating high stability. From the convergence curves (Figure A11, Figure A12, Figure A13, Figure A14 and Figure A15) in Appendix E and the box plots (Figure A16, Figure A17, Figure A18, Figure A19 and Figure A20) in Appendix F, the ISWO algorithm showcases outstanding performance on both simple and complex test functions, reflecting its broad applicability.

In Figure A11, Figure A14 and Figure A15, functions F6 and F30 may contain complex gradient variations, while F8 and F23 may have a large number of local extrema. Other algorithms might possess stronger adaptability when handling these specific functions. Parameters within the algorithm (such as step size or weight coefficients) may not be optimally tuned for these particular functions, leading to suboptimal performance when addressing such problems. This results in the ISWO algorithm appearing slightly weaker in the later stages of iterations.

## 5. Environmental Model for UAV Path Planning

In this study, we constructed an accurate three-dimensional environmental model for UAV trajectory planning, defining the flight area as a 100 × 100 × 250 rectangular space. Gaussian function models [26,27] were employed to simulate obstacles (such as mountains); these not only accurately reproduce terrain undulations but also can be adapted to different geographical environments.

(44) $$z(x,y)=\overset{N}{\underset{i=1}{\sum }}h_{i}(x,y)=\overset{N}{\underset{i=1}{\sum }}H_{i}\exp\left(-\left(\frac{{\left(x-x_{i}\right)}^{2}}{2\sigma_{x_{i}}^{2}}+\frac{{\left(y-y_{i}\right)}^{2}}{2\sigma_{y_{i}}^{2}}\right)\right)$$

**Figure 11:**
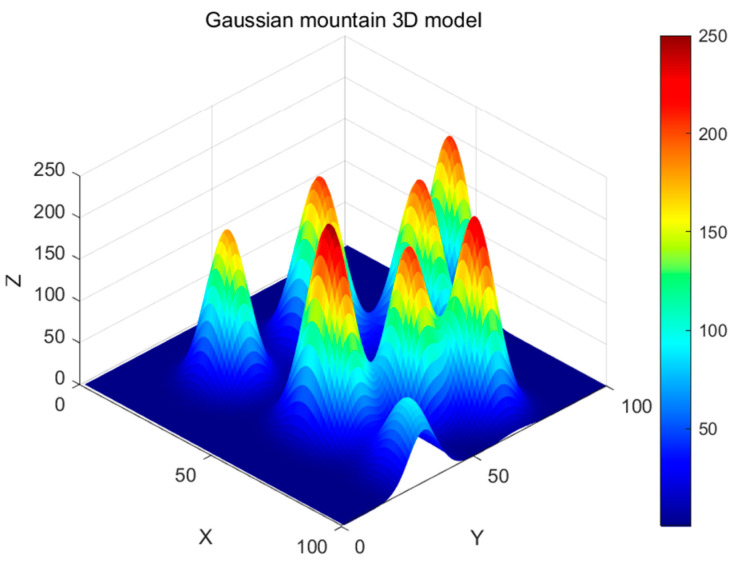
Three-dimensional model of Gaussian mountains.

In Equation (44), *N* represents the total number of mountain peaks, z(x,y) denotes the terrain elevation at the horizontal coordinates (x,y), and $h_{i}(x,y)$ signifies the height of the i-th peak at position (x,y). As illustrated in Figure 11, we randomly determine each peak’s central position, height, and extent:

$\left(x_{i},y_{i}\right)$ are the central coordinates of the i-th peak within the map boundaries.

$H_{i}$ denotes the height of the i-th peak.

$\sigma_{x_{i}},\sigma_{y_{i}}$ control the slope by adjusting the rate of change of the peak along the x- and y-axes, respectively.

## 6. Flight Path and Smoothing Processing

A cubic spline fitting algorithm can be utilized to generate paths and plot three-dimensional surfaces as follows. Begin by initializing the starting point, ending point, and surface coordinates, then merge these points into a sequence. Employ MATLAB’s spline function to perform cubic spline interpolation, thereby generating a smooth path. Use surf(X, Y, Z) to plot the surface graph, apply shading flat to remove grid lines, and set colors with colormap, thus rendering a smooth flight path.

In the Improved Spider Wasp Optimizer algorithm, each path consists of a starting point, an ending point, and waypoint nodes. By optimizing the positions of these waypoints, smooth cubic spline curves are generated through interpolation between adjacent points. The i-th segment of the path contains *n* control points, defined as $f(x_{0})$, $f(x_{1})$, …, $f(x_{n})$, with the domain $x_{0}<x_{1}<x_{2}<\ldots <x_{n}$. Cubic spline interpolation represents the function between adjacent points, ensuring that the function and its first and second derivatives are continuous within the interval, as shown in Equation (45).

(45) $$f_{n}(x)=a_{n}{\left(x-x_{n}\right)}^{3}+b_{n}{\left(x-x_{n}\right)}^{2}+c_{n}(x-x_{n})+d_{n}$$

The *n* polynomial segments require solving 4*n* parameters $a_{n},b_{n},c_{n},d_{n}$ [28]. Based on the continuity of derivatives and interpolation, 4n−2 equations can be derived, as shown in Equation (46):

(46) $$\left\{\begin{array}{l} f_{n}(x_{n})\\ f_{n}(x_{n+1})\\ f_{n}^{\prime }(x_{n+1})\\ f_{n}^{\prime \prime }(x_{n+1}) \end{array}\right.$$

**Figure 12:**
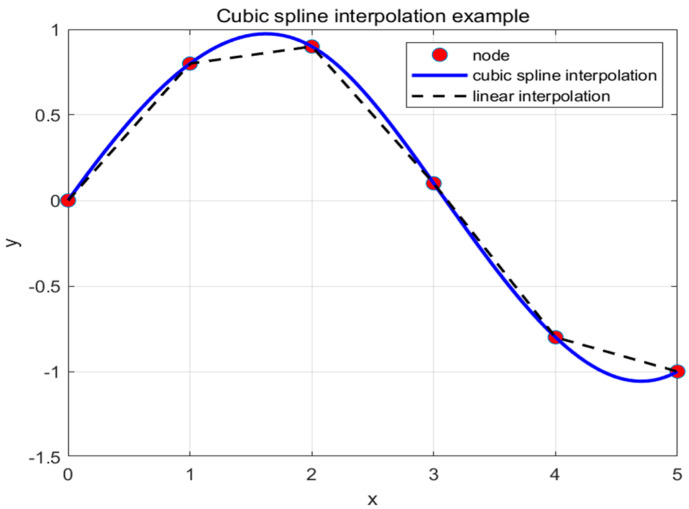
Three times cubic spline interpolation optimizer effect diagram.

**Figure 13:**
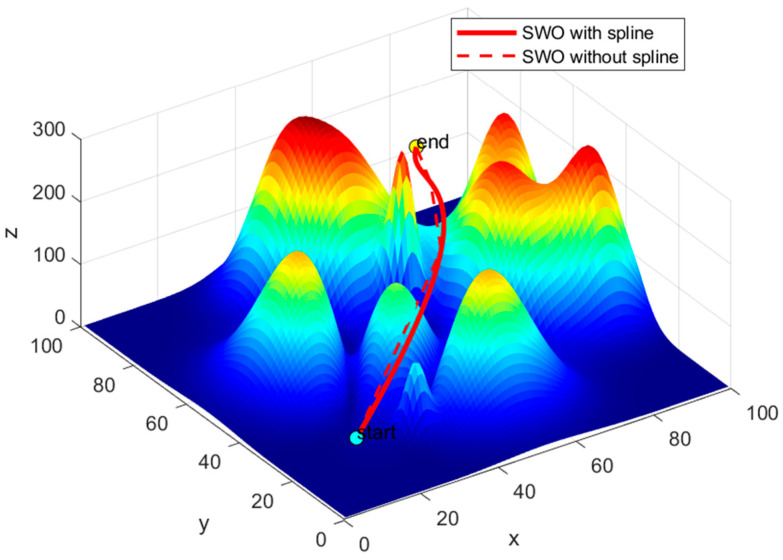
Comparison chart of SWO algorithm curves with and without cubic spline interpolation.

The remaining two conditions are determined by the starting point $x_{0}$ and the target point $x_{n}$. Upon completing the path planning, the UAV will generate a continuous and smooth cubic spline curve. The optimization effect of the cubic spline interpolation is illustrated in Figure 12. By comparing the trajectories with and without the cubic spline in Figure 13, it is evident that the path without the cubic spline is not smooth and is more prone to contacting obstacles.

## 7. Constraint Conditions

To ensure the UAV operates within the specified airspace, its position updates during each iteration must satisfy the following conditions. Boundary constraint condition: The UAV’s position must remain within the upper and lower bounds of the search space. For each dimension j(j=1,2,…,dim), the position $x_{j}$ must satisfy:

(47) $${\mathrm{lb}}_{j}\leq x_{j}\leq {\mathrm{ub}}_{j}$$

In Equation (47), ${lb}_{j}$ and ${ub}_{j}$ represent the lower and upper bounds of the j-th dimension, respectively. Furthermore, to ensure that the flight path remains within the designated airspace, boundary constraints must be applied to satisfy the conditions specified in Equation (48):

(48) $$\left\{\begin{array}{l} 0\leq x_{i}\leq x_{\max}\\ 0\leq y_{i}\leq y_{\max}\\ 0\leq z_{i}\leq z_{\max} \end{array}\right.,\;i=1,2,\ldots ,n$$

The fitness function [29] yields the minimum cost of flight within the designated airspace while avoiding obstacles. It is derived from the objective function expression, as shown in Equation (49):

(49) $$fitness=\min(V_{c}+T_{c}+E_{c})$$

## 8. Objective Function

The objective function for the UAV flight path primarily consists of three key factors: the total flight distance, the obstacle avoidance cost, and constraints to ensure the UAV remains within specified boundaries. This objective function [30,31] considers the following aspects:

(50) $$fitness=V_{c}+T_{c}+E_{c}$$

In Formula (50), $V_{C}$ represents the total voyage cost of the UAV. $T_{c}$ denotes the cost incurred by the UAV when bypassing obstacles. $E_{c}$ signifies the cost of the UAV flying within the specified boundaries.

The voyage cost $V_{c}$ primarily considers the total flight distance of the UAV from the starting point to the endpoint, which is the sum of each arc segment $L_{i}$. If the entire flight path comprises *n* segments, the total voyage cost is expressed as

(51) $$\left\{\begin{array}{l} V_{c}=\overset{n-1}{\underset{i=1}{\sum }}L_{i}\\ L_{i}=\sqrt{{\left(x_{i+1}-x_{i}\right)}^{2}+{\left(y_{i+1}-y_{i}\right)}^{2}+{\left(z_{i+1}-z_{i}\right)}^{2}} \end{array}\right.$$

The terrain cost $T_{c}$ is primarily designed to ensure that the UAV’s flight path avoids obstacles by controlling the value of $T_{c}$. When the altitude $z_{i}$ is higher than the obstacle height $Z_{2}$($x_{i}$, $y_{i}$) in the local terrain, we set $T_{c}$ = 0; when the altitude $z_{i}$ is lower than the obstacle height $Z_{2}$($x_{i}$, $y_{i}$), we set $T_{c}$ = ∞. Summing up in this manner guarantees that the UAV’s flight path can avoid obstacles such as mountains. The expression is as follows:

(52) $$\left\{\begin{array}{l} T_{c}=\overset{n}{\underset{i=1}{\sum }}T_{c_{i}}\;T_{c_{0}}=0\\ T_{c_{i}}=\left\{\begin{array}{l} 0\;Z_{i}>Z\left(x_{i},y_{i}\right)\\ \infty \;otherwise\; \end{array}\right. \end{array}\right.$$

The boundary cost $E_{c}$ is intended to ensure that the UAV remains within the specified airspace, primarily achieved by controlling the value of $E_{c}$. When the UAV is within the airspace, $E_{c}$ = 0; when it is outside the airspace, $E_{c}$ = ∞. Summing in this way guarantees that the UAV’s flight path stays within the designated airspace. The expression is as follows:

(53) $$\left\{\begin{array}{l} E_{c}=\overset{n}{\underset{i=1}{\sum }}E_{c_{i}}E_{c_{0}}=0\\ E_{c_{i}}=\left\{\begin{array}{l} \infty \;otherwise\\ 0\;x_{i}\in \left[0,x_{\max}\right]\cap y_{i}\in [0,y_{\max}]\cap z_{i}\in \left[0,z_{\max}\right] \end{array}\right. \end{array}\right.$$

## 9. Simulation Results Analysis of the ISWO Algorithm and Other Intelligent Algorithms

To validate the effectiveness of the ILO algorithm in simulating three-dimensional UAV paths over mountainous terrain, a complex experimental environment map was established, with the relevant environmental parameters detailed in Table 3. Moreover, the data presented in Table 4 were obtained by averaging the results from 30 trials; the compared algorithms are among the latest intelligent algorithms introduced in 2024.

Based on Table 4, the average convergence iteration count for ISWO is 32, which is significantly less than HO (57), BKA (91), SWO (90), and COA (72). Although CFOA converges in fewer iterations (11 times), its solution quality is inferior. This demonstrates that ISWO achieves efficient convergence by integrating Differential Evolution (DE), COA’s adaptive update mechanisms, and the opposition-based learning (OBL) strategy to enhance the balance between exploration and exploitation.

**Figure 14:**
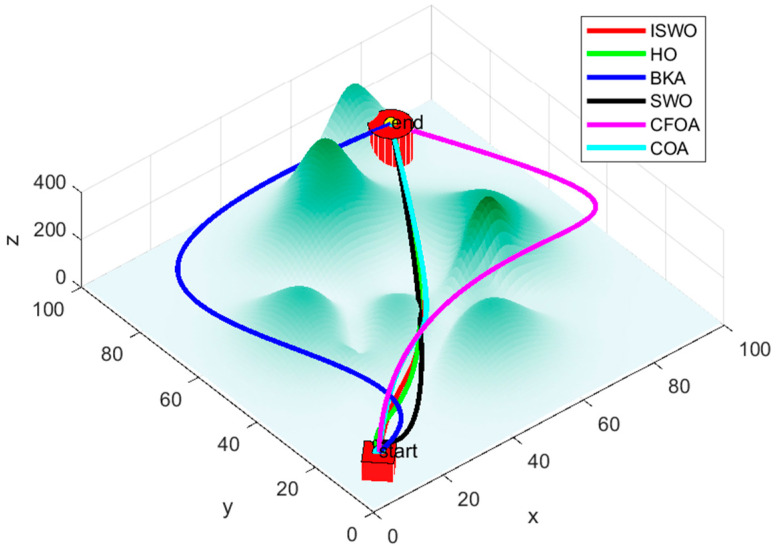
Six algorithms, ISWO, HO, BKA, SWO, CFOA, and COA, for planning routes.

**Figure 15:**
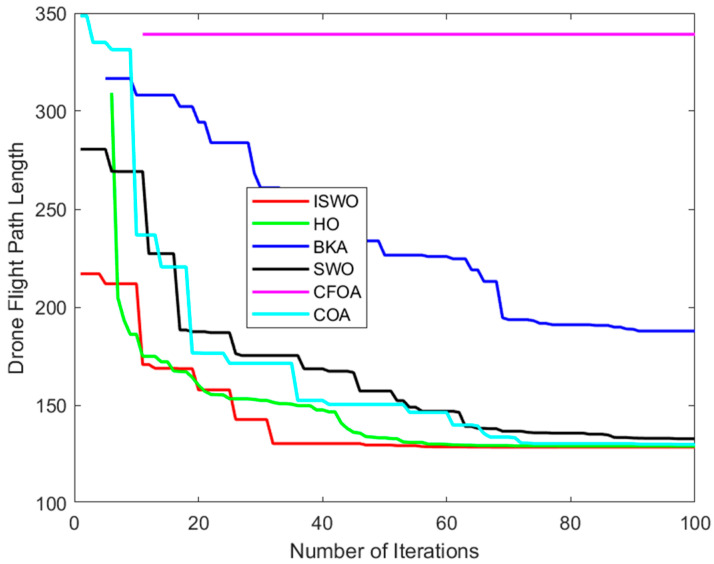
Convergence curves of the six algorithms: ISWO, HO, BKA, SWO, CFOA, and COA.

**Figure 16:**
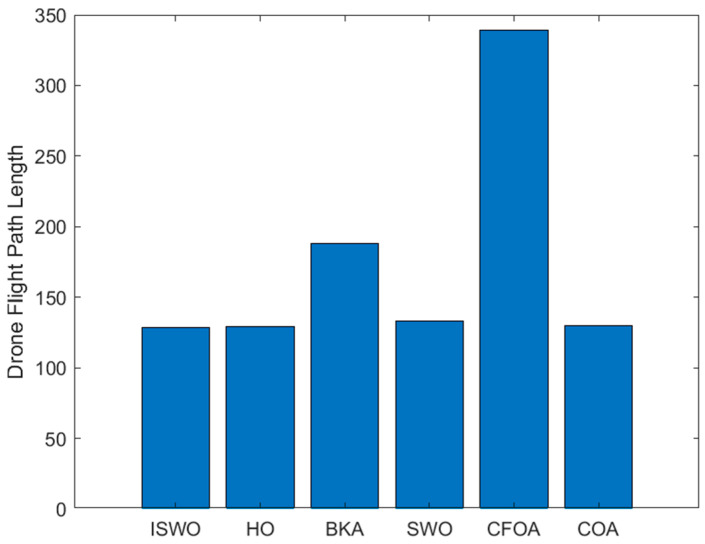
Histogram of the path lengths of the six algorithms: ISWO, HO, BKA, SWO, CFOA, and COA.

From Figure 14, Figure 15 and Figure 16, it is evident that CFOA has the longest average path length (339.2), highlighting its inefficiency. BKA also exhibits a relatively high average path length of 187.8. In contrast, ISWO identifies shorter and more optimal paths, thereby reducing UAV energy consumption and flight time. ISWO dynamically adjusts its exploration and exploitation phases during iterations, enabling it to converge more rapidly while maintaining solution quality. Conversely, other algorithms like CFOA and SWO tend to become trapped in suboptimal solutions or converge slowly due to an imbalance between exploration and exploitation.

The path generated by ISWO is relatively the most direct, with a shorter and smoother trajectory from the “start point” to the “end point”, effectively avoiding obstacles along the shortest possible route and selecting the optimal path to achieve the best outcome. While HO’s path is also relatively effective, it is slightly more curved compared to ISWO, indicating a marginally lesser optimization capability. BKA’s path is noticeably longer than those of ISWO and HO, involving more detours in areas with significant terrain undulations, resulting in a more complex route. This reflects certain limitations of BKA in finding the shortest path and the difficulty it has in effectively avoiding complex terrain features.

Table 5 compares several popular algorithms from recent years. According to the data, the average convergence iteration count for ISWO is 22, which is the lowest among all algorithms and significantly superior to the others. For example, DBO requires 96 iterations, LO requires 98, and SWO also requires 98, indicating that ISWO has made significant improvements in the efficiency of finding the optimal solution. The fitness and convergence rates of other algorithms are also inferior to ISWO. For instance, DBO has a fitness of 96.5% and LO has a fitness of 90.4%, suggesting that these algorithms may become trapped in suboptimal solutions during the solving process.

**Figure 17:**
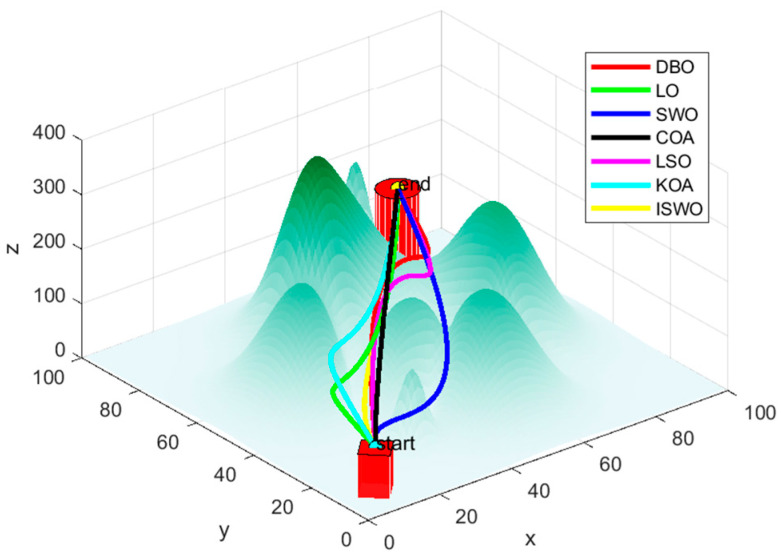
Seven algorithms, DBO, LO, SWO, COA, LSO, KOA, and ISWO, for planning routes.

**Figure 18:**
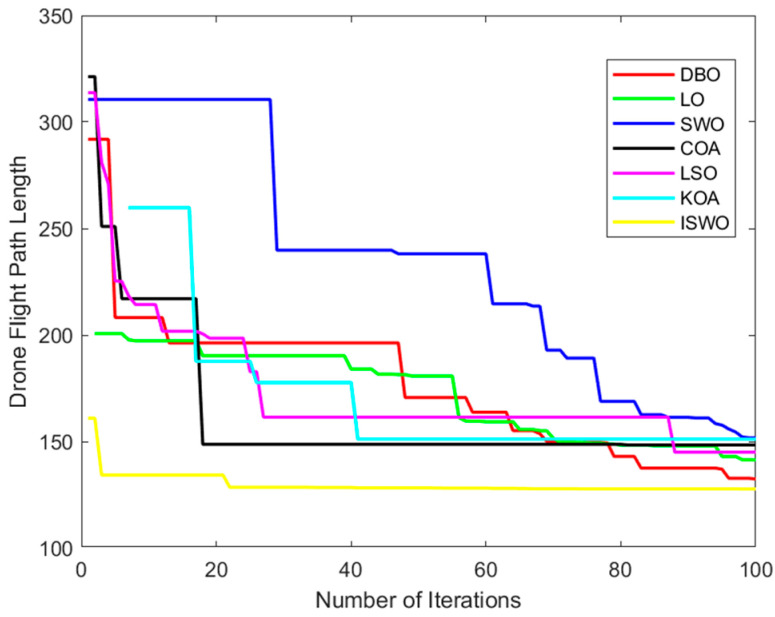
Convergence curves of the six algorithms: DBO, LO, SWO, COA, LSO, KOA, and ISWO.

**Figure 19:**
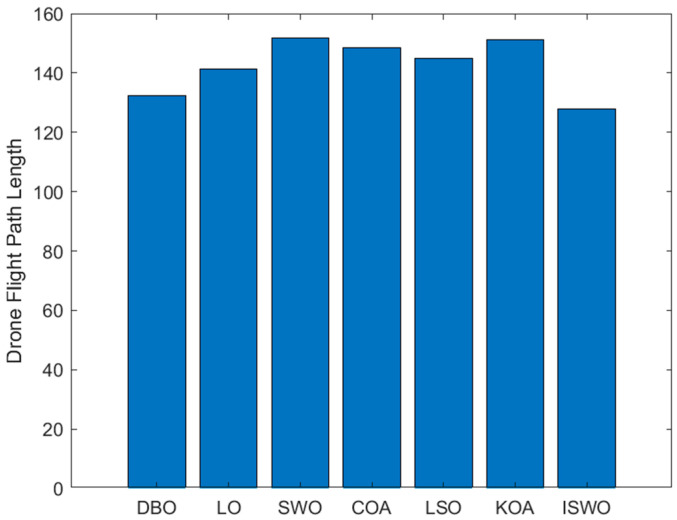
Histogram of the path lengths of the six algorithms: ISWO, HO, BKA, SWO, CFOA, and COA.

From Figure 17, it can be observed that the paths generated by LO, SWO, and COA involve more detours or are more complex, whereas ISWO is able to find shorter and more direct paths. This implies that ISWO performs better in optimizing UAV flight paths and can significantly reduce flight distance and energy consumption. Figure 18 shows that the ISWO algorithm can find the optimal path more quickly, with a convergence speed significantly faster than other algorithms, thereby saving computation time. Figure 19 indicates that the average path length of ISWO is approximately 127.7, while other algorithms such as SWO, KOA, and COA have average path lengths exceeding 140. This demonstrates that ISWO can find significantly better paths. Such shorter path lengths directly reflect the superior optimization performance of ISWO, making UAV flight more efficient.

## 10. Concluding Remarks

This paper introduces an Improved Spider Wasp Optimizer (ISWO), which innovatively modifies the population iteration formula (25) of the original SWO algorithm to resolve inaccuracies in computing N during each iteration. By integrating the strengths of Differential Evolution (DE) and the Crayfish Optimization Algorithm (COA), and by incorporating the opposition-based learning (OBL) strategy, the ISWO achieves dynamic adaptive parameter balancing between the exploration and exploitation phases. This significantly enhances the algorithm’s global search capability and convergence speed.

The experimental results indicate that ISWO surpasses other comparative algorithms in terms of minimum value, mean value, and standard deviation across 50-dimensional and 100-dimensional test functions, demonstrating superior stability and robustness. In UAV path planning applications, ISWO is capable of generating shorter and smoother flight paths with fewer iterations, thereby reducing energy consumption and flight time.

The opposition-based learning strategy is applied once every 10 iterations. In high-dimensional complex problems, this low-frequency application may not effectively maintain population diversity, especially during the early iterations when the population may quickly converge to certain local regions, lacking sufficient diversity to explore the global search space. Consequently, ISWO exhibits certain limitations when handling highly complex multimodal functions (such as F30) and functions requiring fine-grained exploitation (such as F5 and F6). It tends to become trapped in local optima, affecting the efficiency of global optimum search. Future research could consider introducing new mechanisms, such as strategies to enhance population diversity, dynamic parameter adjustments, or hybridization with other optimization algorithms, to further improve ISWO’s performance on complex optimization problems. Overall, ISWO demonstrates exceptional advantages in both algorithmic performance and practical applications. It adapts well to complex terrains and provides efficient and reliable solutions, yet there remains room for improvement to enhance its optimization capabilities on intricate functions.

## Figures and Tables

**Table 1:** Summary of the CEC’17 test functions [25].

	No.	Function	Fi* = *Fi*(x*)
Unimodal Functions	1	Shifted and Rotated Bent Cigar Function	100
	2	Shifted and Rotated Sum of Different Power Function	200
	3	Shifted and Rotated Zakharov Function	300
Simple Multimodal Functions	4	Shifted and Rotated Rosenbrock’s Function	400
	5	Shifted and Rotated Rastrigin’s Function	500
	6	Shifted and Rotated Expanded Scaffer’s F6 Function	600
	7	Shifted and Rotated Lunacek Bi_Rastrigin Function	700
	8	Shifted and Rotated Non-Continuous Rastrigin’s Function	800
	9	Shifted and Rotated Lévy Function	900
	10	Shifted and Rotated Schwefel’s Function	1000
Hybrid Functions	11	Hybrid Function 1 (*N* = 3)	1100
	12	Hybrid Function 2 (*N* = 3)	1200
	13	Hybrid Function 3 (*N* = 3)	1300
	14	Hybrid Function 4 (*N* = 4)	1400
	15	Hybrid Function 5 (*N* = 4)	1500
	16	Hybrid Function 6 (*N* = 4)	1600
	17	Hybrid Function 6 (*N* = 5)	1700
	18	Hybrid Function 6 (*N* = 5)	1800
	19	Hybrid Function 6 (*N* = 5)	1900
	20	Hybrid Function 6 (*N* = 6)	2000
Composition Functions	21	Composition Function 1 (*N* = 3)	2100
	22	Composition Function 2 (*N* = 3)	2200
	23	Composition Function 3 (*N* = 4)	2300
	24	Composition Function 4 (*N* = 4)	2400
	25	Composition Function 5 (*N* = 5)	2500
	26	Composition Function 6 (*N* = 5)	2600
	27	Composition Function 7 (*N* = 6)	2700
	28	Composition Function 8 (*N* = 6)	2800
	29	Composition Function 9 (*N* = 3)	2900
	30	Composition Function 10 (*N* = 3)	3000
Search Range: ${\left[-100,100\right]}^{D}$			

**Table 2:** Algorithm parameters.

Algorithm	Population	Number of Iterations	Parameters
HO	30	500	*t* = 1
BKA	30	500	*p* = 0.9, *r = rand*
CFOA	30	500	Efs=0
SWO	30	500	TR=0.3, Cr=0.2,t=0
COA	30	500	*t* = 1, C = 2
ISWO	30	500	TR=0.3, Cr=0.9, F=0.5

**Table 3:** Environmental parameters.

	Parameter	Notation	Parameter Value
Map	Execution space (math.)		100 × 100 × 250
	Starting point	Start	[10, 10, 80]
	Target point	Goal	[80, 90, 150]
	Number of peaks	N	5
	Population size	SearchAgents_no	30
	Number of iterations	t	100

**Table 4:** Comparison of average fitness value and convergence speed.

Scale	Algorithm	Average Number of Convergence Iterations	Mean Fitness Value	Percentage of ISWO Adaptation Values/%	Percentage of ISWO Converged Iterations/%
Map	ISWO	32	128.5	100	100
	HO	57	129.1	99.5	56.1
	BKA	91	187.8	68.4	35.2
	SWO	90	132.8	96.8	35.6
	CFOA	11	339.2	37.9	
	COA	72	129.9	98.9	44.5

**Table 5:** Comparison of average fitness value and convergence speed.

Scale	Algorithm	Average Number of Convergence Iterations	Mean Fitness Value	Percentage of ISWO Adaptation Values/%	Percentage of ISWO Converged Iterations/%
Map	DBO	96	132.4	96.5	22.9
	LO	98	141.4	90.4	22.4
	SWO	98	151.7	68.4	22.4
	COA	81	148.4	84.2	27.2
	LSO	88	145.0	88.1	25
	KOA	41	151.1	84.5	53.7
	ISWO	22	127.7	100	100

## Data Availability

The raw data supporting the conclusions of this article will be made available by the authors on request.

## Appendix A

**Table A1:** Comparison of 6 algorithms of 50 dimensions.

		Dim = 50					
		HO	BKA	CFOA	SWO	COA	ISWO
F1	Max	1.81E+10	1.03E+11	1.40E+11	7.78E+08	2.42E+10	1.39E+09
	Mean	1.07E+10	4.25E+10	1.06E+11	4.27E+08	1.14E+10	2.61E+08
	Min	4.51E+09	1.54E+10	8.55E+10	1.90E+08	3.54E+09	5.64E+07
	*p*-value	3.02E−11	3.02E−11	3.02E−11	2.15E−06	3.02E−11	1.00E+00
	Std	3.63E+09	2.26E+10	1.26E+10	1.56E+08	5.66E+09	2.68E+08
F2	Max	1.26E+66	6.96E+75	1.38E+87	2.84E+48	1.63E+61	2.98E+49
	Mean	6.84E+64	2.42E+74	5.39E+85	1.23E+47	5.43E+59	1.93E+48
	Min	9.37E+54	2.19E+46	1.33E+70	5.65E+38	1.02E+42	4.50E+37
	*p*-value	3.02E−11	9.92E−11	3.02E−11	1.63E−02	1.20E−08	1.00E+00
	Std	2.46E+65	1.27E+75	2.52E+86	5.20E+47	2.97E+60	7.33E+48
F3	Max	1.62E+05	2.26E+05	7.82E+05	1.91E+05	3.00E+05	1.29E+05
	Mean	1.40E+05	9.41E+04	4.67E+05	1.20E+05	2.35E+05	8.95E+04
	Min	1.20E+05	5.34E+04	3.15E+05	7.86E+04	1.66E+05	6.60E+04
	*p*-value	4.98E−11	6.63E−01	3.02E−11	2.32E−06	3.02E−11	1.00E+00
	Std	1.14E+04	3.67E+04	1.12E+05	2.48E+04	3.40E+04	1.51E+04
F4	Max	3.57E+03	3.06E+04	4.34E+04	8.54E+02	4.49E+03	8.79E+02
	Mean	2.28E+03	9.16E+03	2.95E+04	7.43E+02	2.02E+03	7.39E+02
	Min	1.43E+03	1.75E+03	1.81E+04	5.86E+02	1.03E+03	6.09E+02
	*p*-value	3.02E−11	3.02E−11	3.02E−11	6.52E−01	3.02E−11	1.00E+00
	Std	4.95E+02	8.69E+03	5.62E+03	6.14E+01	8.97E+02	6.64E+01
F5	Max	9.54E+02	1.18E+03	1.30E+03	8.73E+02	9.46E+02	8.71E+02
	Mean	8.93E+02	9.10E+02	1.21E+03	7.51E+02	8.95E+02	8.06E+02
	Min	8.39E+02	7.79E+02	1.08E+03	6.76E+02	8.20E+02	7.09E+02
	*p*-value	2.87E−10	9.76E−10	3.02E−11	2.96E−05	2.15E−10	1.00E+00
	Std	2.83E+01	8.19E+01	4.78E+01	4.21E+01	2.54E+01	4.32E+01
F6	Max	6.85E+02	7.04E+02	7.18E+02	6.27E+02	6.80E+02	6.56E+02
	Mean	6.75E+02	6.74E+02	7.01E+02	6.17E+02	6.66E+02	6.43E+02
	Min	6.56E+02	6.63E+02	6.80E+02	6.09E+02	6.38E+02	6.28E+02
	*P*-value	3.02E−11	3.02E−11	3.02E−11	3.02E−11	2.23E−09	1.00E+00
	Std	5.78E+00	1.01E+01	8.10E+00	4.58E+00	9.02E+00	7.32E+00
F7	Max	1.84E+03	1.92E+03	2.86E+03	1.28E+03	1.85E+03	1.53E+03
	Mean	1.69E+03	1.71E+03	2.51E+03	1.14E+03	1.76E+03	1.29E+03
	Min	1.41E+03	1.57E+03	1.95E+03	1.01E+03	1.47E+03	1.05E+03
	*p*-value	4.98E−11	3.02E−11	3.02E−11	2.68E−06	4.50E−11	1.00E+00
	Std	7.84E+01	6.81E+01	2.26E+02	7.38E+01	8.28E+01	1.19E+02
F8	Max	1.24E+03	1.47E+03	1.60E+03	1.13E+03	1.30E+03	1.22E+03
	Mean	1.16E+03	1.26E+03	1.51E+03	1.06E+03	1.23E+03	1.11E+03
	Min	1.04E+03	1.18E+03	1.38E+03	9.72E+02	1.07E+03	1.00E+03
	*p*-value	4.35E−05	1.96E−10	3.02E−11	1.30E−03	8.89E−10	1.00E+00
	Std	4.23E+01	7.35E+01	5.75E+01	4.28E+01	4.05E+01	5.33E+01
F9	Max	2.40E+04	3.58E+04	6.57E+04	1.39E+04	3.64E+04	2.17E+04
	Mean	1.99E+04	1.76E+04	4.64E+04	7.52E+03	2.51E+04	1.31E+04
	Min	8.51E+03	1.38E+04	2.91E+04	3.47E+03	1.40E+04	6.53E+03
	*p*-value	2.20E−07	4.46E−04	3.02E−11	1.73E−07	1.20E−08	1.00E+00
	Std	3.17E+03	4.58E+03	1.08E+04	2.22E+03	6.87E+03	4.35E+03
F10	Max	1.25E+04	1.47E+04	1.72E+04	1.20E+04	1.47E+04	1.04E+04
	Mean	9.69E+03	9.37E+03	1.59E+04	9.39E+03	1.34E+04	8.43E+03
	Min	7.82E+03	6.78E+03	1.35E+04	7.45E+03	1.11E+04	6.57E+03
	*p*-value	1.49E−04	3.78E−02	3.02E−11	5.83E−03	3.02E−11	1.00E+00
	Std	1.28E+03	1.97E+03	7.89E+02	1.31E+03	7.29E+02	8.94E+02
F11	Max	7.09E+03	2.77E+04	6.64E+04	2.50E+03	1.27E+04	2.15E+03
	Mean	4.78E+03	5.42E+03	4.63E+04	1.80E+03	4.81E+03	1.65E+03
	Min	3.54E+03	2.00E+03	2.57E+04	1.49E+03	2.04E+03	1.33E+03
	*p*-value	3.02E−11	4.98E−11	3.02E−11	7.29E−03	4.08E−11	1.00E+00
	Std	1.04E+03	4.97E+03	1.30E+04	2.30E+02	2.47E+03	1.92E+02
F12	Max	2.82E+09	4.81E+10	9.14E+10	5.67E+07	6.87E+09	1.76E+08
	Mean	1.22E+09	6.63E+09	6.20E+10	2.68E+07	7.50E+08	2.49E+07
	Min	1.93E+08	5.29E+07	2.41E+10	6.95E+06	5.72E+07	2.82E+06
	*p*-value	3.02E−11	3.69E−11	3.02E−11	6.35E−02	1.33E−10	1.00E+00
	Std	6.71E+08	1.11E+10	1.50E+10	1.38E+07	1.51E+09	3.14E+07
F13	Max	1.44E+08	2.74E+10	5.26E+10	5.35E+04	7.76E+06	1.20E+05
	Mean	7.28E+06	2.37E+09	2.89E+10	2.06E+04	1.88E+06	2.87E+04
	Min	2.05E+05	4.36E+05	6.34E+09	7.70E+03	1.04E+05	1.26E+04
	*p*-value	3.02E−11	3.02E−11	3.02E−11	3.92E−02	3.34E−11	1.00E+00
	Std	2.72E+07	6.40E+09	1.18E+10	1.03E+04	2.14E+06	2.01E+04
F14	Max	7.44E+06	1.30E+07	1.31E+08	6.28E+05	4.39E+06	1.30E+05
	Mean	2.90E+06	5.94E+05	3.68E+07	1.69E+05	1.48E+06	4.54E+04
	Min	4.18E+05	1.18E+04	4.40E+06	6.54E+03	1.09E+05	4.93E+03
	*p*-value	3.02E−11	8.66E−05	3.02E−11	1.41E−04	3.69E−11	1.00E+00
	Std	2.29E+06	2.36E+06	2.81E+07	1.60E+05	1.09E+06	3.60E+04
F15	Max	2.44E+05	5.10E+09	9.80E+09	2.67E+04	1.00E+06	2.33E+04
	Mean	9.03E+04	4.18E+08	4.30E+09	9.28E+03	9.30E+04	1.19E+04
	Min	2.90E+04	3.72E+04	9.83E+08	2.50E+03	9.12E+03	3.18E+03
	*p*-value	3.02E−11	3.02E−11	3.02E−11	9.33E−02	3.82E−10	1.00E+00
	Std	5.66E+04	1.09E+09	2.35E+09	6.24E+03	1.78E+05	6.47E+03
F16	Max	8.18E+03	8.90E+03	1.05E+04	4.13E+03	4.62E+03	4.42E+03
	Mean	5.41E+03	4.61E+03	7.78E+03	3.44E+03	3.72E+03	3.40E+03
	Min	3.96E+03	3.06E+03	5.71E+03	2.71E+03	2.53E+03	2.76E+03
	*p*-value	4.98E−11	4.44E−07	3.02E−11	3.95E−01	9.47E−03	1.00E+00
	Std	8.47E+02	1.35E+03	1.30E+03	3.32E+02	5.08E+02	4.18E+02
F17	Max	4.67E+03	5.16E+03	3.19E+04	3.57E+03	4.35E+03	3.51E+03
	Mean	3.84E+03	3.69E+03	1.01E+04	2.97E+03	3.59E+03	3.07E+03
	Min	3.07E+03	3.03E+03	4.65E+03	2.50E+03	2.92E+03	2.47E+03
	*p*-value	3.50E−09	3.50E−09	3.02E−11	2.28E−01	6.01E−08	1.00E+00
	Std	4.47E+02	4.69E+02	6.72E+03	2.62E+02	3.64E+02	2.18E+02
F18	Max	1.83E+07	7.01E+07	4.14E+08	5.38E+06	2.38E+07	2.78E+06
	Mean	6.18E+06	5.19E+06	1.12E+08	1.48E+06	6.51E+06	5.97E+05
	Min	3.39E+05	1.59E+05	2.08E+07	1.47E+05	6.70E+05	6.62E+04
	*p*-value	3.47E−10	9.03E−04	3.02E−11	1.25E−04	4.20E−10	1.00E+00
	Std	4.21E+06	1.43E+07	8.60E+07	1.21E+06	4.65E+06	5.91E+05
F19	Max	3.52E+07	1.78E+09	6.77E+09	4.15E+04	9.89E+05	3.12E+04
	Mean	1.17E+07	9.67E+07	2.23E+09	1.77E+04	2.40E+05	1.49E+04
	Min	5.50E+04	8.65E+04	5.13E+08	2.06E+03	3.45E+04	2.70E+03
	*p*-value	3.02E−11	3.02E−11	3.02E−11	3.63E−01	3.02E−11	1.00E+00
	Std	1.06E+07	3.42E+08	1.35E+09	1.12E+04	2.17E+05	7.75E+03
F20	Max	3.78E+03	3.85E+03	5.29E+03	3.65E+03	3.93E+03	3.58E+03
	Mean	3.30E+03	3.27E+03	4.47E+03	3.01E+03	3.63E+03	3.04E+03
	Min	2.91E+03	2.74E+03	3.45E+03	2.43E+03	2.79E+03	2.52E+03
	*p*-value	2.53E−04	4.64E−03	3.69E−11	8.07E−01	7.09E−08	1.00E+00
	Std	1.97E+02	2.76E+02	4.82E+02	2.43E+02	3.17E+02	2.71E+02
F21	Max	3.02E+03	3.28E+03	3.34E+03	2.61E+03	2.74E+03	2.62E+03
	Mean	2.83E+03	2.89E+03	3.11E+03	2.54E+03	2.61E+03	2.53E+03
	Min	2.68E+03	2.73E+03	2.96E+03	2.48E+03	2.50E+03	2.44E+03
	*p*-value	3.02E−11	3.02E−11	3.02E−11	3.87E−01	6.28E−06	1.00E+00
	Std	8.40E+01	1.19E+02	9.51E+01	4.17E+01	6.67E+01	4.71E+01
F22	Max	1.39E+04	1.60E+04	1.89E+04	1.39E+04	1.66E+04	1.35E+04
	Mean	1.18E+04	1.13E+04	1.74E+04	1.07E+04	1.45E+04	8.06E+03
	Min	8.88E+03	9.73E+03	1.55E+04	2.83E+03	4.99E+03	2.43E+03
	*p*-value	8.20E−07	1.43E−05	3.02E−11	1.17E−03	4.20E−10	1.00E+00
	Std	1.19E+03	1.39E+03	8.75E+02	1.82E+03	2.16E+03	3.64E+03
F23	Max	3.98E+03	4.35E+03	4.40E+03	3.17E+03	3.51E+03	3.27E+03
	Mean	3.59E+03	3.86E+03	4.16E+03	3.06E+03	3.22E+03	3.10E+03
	Min	3.38E+03	3.36E+03	3.85E+03	2.96E+03	3.09E+03	2.98E+03
	*p*-value	3.02E−11	3.02E−11	3.02E−11	2.61E−02	2.03E−07	1.00E+00
	Std	1.46E+02	2.54E+02	1.64E+02	5.67E+01	8.93E+01	6.74E+01
F24	Max	4.27E+03	4.17E+03	4.71E+03	3.35E+03	3.66E+03	3.46E+03
	Mean	3.88E+03	3.83E+03	4.29E+03	3.24E+03	3.43E+03	3.29E+03
	Min	3.53E+03	3.46E+03	3.81E+03	3.13E+03	3.22E+03	3.14E+03
	*p*-value	3.02E−11	3.34E−11	3.02E−11	4.64E−03	5.61E−05	1.00E+00
	Std	1.75E+02	1.57E+02	2.05E+02	6.16E+01	1.27E+02	8.06E+01
F25	Max	5.20E+03	1.55E+04	2.46E+04	3.51E+03	4.69E+03	3.50E+03
	Mean	4.28E+03	6.95E+03	1.58E+04	3.28E+03	4.06E+03	3.28E+03
	Min	3.63E+03	4.32E+03	9.97E+03	3.15E+03	3.50E+03	3.15E+03
	*p*-value	3.02E−11	3.02E−11	3.02E−11	7.39E−01	3.34E−11	1.00E+00
	Std	4.18E+02	3.42E+03	2.93E+03	9.70E+01	3.57E+02	9.20E+01
F26	Max	1.38E+04	1.83E+04	2.06E+04	8.53E+03	1.38E+04	1.16E+04
	Mean	1.21E+04	1.34E+04	1.78E+04	7.28E+03	1.15E+04	6.53E+03
	Min	8.08E+03	1.06E+04	1.47E+04	6.24E+03	7.10E+03	3.60E+03
	*p*-value	9.76E−10	5.49E−11	3.02E−11	1.19E−01	1.31E−08	1.00E+00
	Std	1.27E+03	1.88E+03	1.65E+03	5.85E+02	1.75E+03	2.65E+03
F27	Max	6.03E+03	5.40E+03	7.11E+03	3.92E+03	4.55E+03	4.13E+03
	Mean	4.54E+03	4.32E+03	5.70E+03	3.73E+03	4.10E+03	3.85E+03
	Min	3.78E+03	3.69E+03	4.39E+03	3.56E+03	3.58E+03	3.64E+03
	*p*-value	9.76E−10	6.53E−07	3.02E−11	4.71E−04	2.32E−06	1.00E+00
	Std	4.45E+02	4.33E+02	6.71E+02	1.14E+02	2.07E+02	1.22E+02
F28	Max	6.08E+03	9.35E+03	1.40E+04	4.07E+03	5.43E+03	3.85E+03
	Mean	5.21E+03	5.98E+03	1.22E+04	3.81E+03	4.60E+03	3.63E+03
	Min	4.16E+03	4.36E+03	9.86E+03	3.48E+03	4.11E+03	3.43E+03
	*p*-value	3.02E−11	3.02E−11	3.02E−11	5.09E−06	3.02E−11	1.00E+00
	Std	4.30E+02	9.30E+02	1.07E+03	1.55E+02	3.51E+02	9.41E+01
F29	Max	1.26E+04	1.84E+04	1.92E+05	5.35E+03	6.09E+03	5.57E+03
	Mean	8.38E+03	7.63E+03	4.43E+04	4.63E+03	5.32E+03	4.86E+03
	Min	6.18E+03	4.96E+03	9.47E+03	3.97E+03	4.87E+03	4.20E+03
	*p*-value	3.02E−11	1.09E−10	3.02E−11	1.50E−02	2.88E−06	1.00E+00
	Std	1.34E+03	2.69E+03	4.72E+04	3.41E+02	2.70E+02	3.41E+02
F30	Max	5.60E+08	1.80E+09	1.01E+10	1.22E+07	9.11E+07	3.88E+07
	Mean	2.85E+08	1.96E+08	4.97E+09	7.02E+06	4.73E+07	1.85E+07
	Min	1.04E+08	3.02E+07	1.55E+09	3.65E+06	1.73E+07	9.72E+06
	*p*-value	3.02E−11	3.69E−11	3.02E−11	1.09E−10	8.89E−10	1.00E+00
	Std	1.29E+08	4.16E+08	2.31E+09	2.12E+06	1.89E+07	6.77E+06

## Appendix B

**Table A2:** Comparison of 6 algorithms of 100 dimensions.

		Dim = 100					
		HO	BKA	CFOA	SWO	COA	ISWO
F1	Max	8.96E+10	1.97E+11	3.27E+11	2.48E+10	1.13E+11	2.77E+10
	Mean	7.48E+10	1.42E+11	2.84E+11	1.78E+10	7.44E+10	1.51E+10
	Min	5.02E+10	1.18E+11	2.23E+11	9.52E+09	5.34E+10	6.66E+09
	*p*-value	3.02E−11	3.02E−11	3.02E−11	6.97E−03	3.02E−11	1.00E+00
	Std	9.22E+09	1.57E+10	2.79E+10	3.43E+09	1.51E+10	4.73E+09
F2	Max	3.74E+159	9.52E+166	3.15E+186	4.74E+133	2.86E+143	3.68E+128
	Mean	1.32E+158	3.18E+165	1.17E+185	1.58E+132	1.04E+142	1.24E+127
	Min	4.37E+138	1.35E+123	4.32E+166	1.27E+113	7.59E+120	8.53E+109
	*p*-value	3.02E−11	6.70E−11	3.02E−11	1.56E−02	1.09E−10	1.00E+00
	Std	Infinity	Infinity	Infinity	8.66E+132	5.22E+142	6.71E+127
F3	Max	3.35E+05	4.52E+05	1.65E+06	3.91E+05	7.38E+05	3.27E+05
	Mean	3.17E+05	2.83E+05	1.03E+06	3.30E+05	5.35E+05	2.74E+05
	Min	2.85E+05	2.25E+05	5.65E+05	2.76E+05	3.96E+05	1.99E+05
	*p*-value	4.31E−08	9.00E−01	3.02E−11	5.09E−08	3.02E−11	1.00E+00
	Std	1.46E+04	4.78E+04	2.87E+05	3.34E+04	8.14E+04	2.86E+04
F4	Max	1.53E+04	7.16E+04	1.40E+05	3.59E+03	2.29E+04	4.68E+03
	Mean	1.23E+04	2.57E+04	9.50E+04	2.68E+03	1.12E+04	2.61E+03
	Min	9.63E+03	1.32E+04	7.37E+04	1.89E+03	5.45E+03	1.53E+03
	*p*-value	3.02E−11	3.02E−11	3.02E−11	3.87E−01	3.02E−11	1.00E+00
	Std	1.38E+03	1.30E+04	1.65E+04	3.99E+02	4.27E+03	6.71E+02
F5	Max	1.64E+03	2.14E+03	2.33E+03	1.54E+03	1.66E+03	1.49E+03
	Mean	1.54E+03	1.60E+03	2.19E+03	1.35E+03	1.53E+03	1.36E+03
	Min	1.47E+03	1.37E+03	2.08E+03	1.15E+03	1.47E+03	1.20E+03
	*p*-value	6.70E−11	1.07E−09	3.02E−11	6.00E−01	1.21E−10	1.00E+00
	Std	4.18E+01	2.06E+02	7.69E+01	9.20E+01	4.91E+01	8.17E+01
F6	Max	6.88E+02	6.96E+02	7.27E+02	6.53E+02	6.80E+02	6.75E+02
	Mean	6.82E+02	6.79E+02	7.15E+02	6.42E+02	6.74E+02	6.65E+02
	Min	6.74E+02	6.69E+02	6.95E+02	6.31E+02	6.69E+02	6.52E+02
	*p*-value	3.34E−11	2.61E−10	3.02E−11	3.34E−11	1.20E−08	1.00E+00
	Std	3.25E+00	4.91E+00	6.64E+00	5.64E+00	2.56E+00	5.97E+00
F7	Max	3.61E+03	4.05E+03	6.05E+03	3.00E+03	3.66E+03	3.10E+03
	Mean	3.43E+03	3.35E+03	5.29E+03	2.41E+03	3.41E+03	2.68E+03
	Min	3.30E+03	3.15E+03	4.60E+03	1.92E+03	3.11E+03	2.30E+03
	*p*-value	3.02E−11	3.02E−11	3.02E−11	3.83E−06	3.02E−11	1.00E+00
	Std	8.75E+01	2.02E+02	3.68E+02	2.08E+02	1.23E+02	1.83E+02
F8	Max	2.10E+03	2.57E+03	2.84E+03	1.82E+03	2.15E+03	1.95E+03
	Mean	1.97E+03	2.06E+03	2.61E+03	1.66E+03	2.03E+03	1.72E+03
	Min	1.75E+03	1.82E+03	2.40E+03	1.48E+03	1.90E+03	1.51E+03
	*p*-value	5.57E−10	1.78E−10	3.02E−11	1.12E−02	3.69E−11	1.00E+00
	Std	8.79E+01	1.91E+02	9.66E+01	8.53E+01	5.40E+01	9.55E+01
F9	Max	5.27E+04	8.84E+04	1.32E+05	7.75E+04	7.33E+04	5.24E+04
	Mean	4.48E+04	4.11E+04	1.00E+05	4.36E+04	4.51E+04	3.77E+04
	Min	3.59E+04	3.11E+04	7.53E+04	2.30E+04	3.24E+04	2.32E+04
	*p*-value	8.66E−05	5.69E−01	3.02E−11	2.46E−01	6.10E−03	1.00E+00
	Std	4.65E+03	1.61E+04	1.40E+04	1.40E+04	1.06E+04	6.98E+03
F10	Max	2.35E+04	3.20E+04	3.57E+04	2.97E+04	3.02E+04	2.13E+04
	Mean	2.13E+04	2.19E+04	3.35E+04	2.36E+04	2.51E+04	1.88E+04
	Min	1.80E+04	1.77E+04	3.11E+04	1.86E+04	1.95E+04	1.49E+04
	*p*-value	7.09E−08	1.89E−04	3.02E−11	2.03E−09	1.61E−10	1.00E+00
	Std	1.25E+03	4.32E+03	1.31E+03	2.50E+03	2.86E+03	1.54E+03
F11	Max	1.41E+05	2.14E+05	1.04E+06	9.54E+04	2.66E+05	6.54E+04
	Mean	1.13E+05	9.08E+04	5.49E+05	5.93E+04	1.75E+05	4.02E+04
	Min	8.72E+04	5.02E+04	3.08E+05	4.12E+04	1.30E+05	1.83E+04
	*p*-value	3.02E−11	1.96E−10	3.02E−11	2.78E−07	3.02E−11	1.00E+00
	Std	1.68E+04	4.62E+04	1.78E+05	1.18E+04	3.50E+04	1.06E+04
F12	Max	2.17E+10	1.82E+11	2.01E+11	2.11E+09	4.23E+10	3.09E+09
	Mean	1.57E+10	5.99E+10	1.61E+11	1.34E+09	1.85E+10	1.11E+09
	Min	8.91E+09	2.27E+10	1.12E+11	8.21E+08	2.99E+09	5.12E+08
	*p*-value	3.02E−11	3.02E−11	3.02E−11	7.96E−03	3.34E−11	1.00E+00
	Std	3.49E+09	3.74E+10	2.39E+10	3.89E+08	1.07E+10	5.46E+08
F13	Max	1.47E+09	4.07E+10	5.34E+10	2.55E+06	3.28E+09	5.13E+06
	Mean	5.06E+08	8.49E+09	3.98E+10	1.08E+06	7.77E+08	8.92E+05
	Min	8.12E+07	1.95E+09	2.93E+10	2.69E+05	3.26E+06	6.90E+04
	*p*-value	3.02E−11	3.02E−11	3.02E−11	1.17E−03	4.08E−11	1.00E+00
	Std	3.45E+08	8.78E+09	6.65E+09	6.21E+05	9.26E+08	1.36E+06
F14	Max	1.75E+07	4.03E+07	1.93E+08	6.15E+06	1.79E+07	2.32E+06
	Mean	8.27E+06	4.80E+06	1.13E+08	2.54E+06	9.94E+06	1.04E+06
	Min	2.61E+06	6.12E+05	3.90E+07	2.82E+05	2.20E+06	2.86E+05
	*p*-value	3.02E−11	1.00E−03	3.02E−11	5.09E−06	3.34E−11	1.00E+00
	Std	3.79E+06	9.24E+06	4.50E+07	1.54E+06	4.25E+06	4.64E+05
F15	Max	6.68E+07	3.13E+10	2.45E+10	8.78E+04	1.52E+09	9.59E+04
	Mean	4.67E+06	2.35E+09	1.73E+10	2.62E+04	9.29E+07	3.03E+04
	Min	1.66E+05	1.37E+06	8.72E+09	1.08E+04	1.47E+05	1.24E+04
	*p*-value	3.02E−11	3.02E−11	3.02E−11	8.77E−02	3.02E−11	1.00E+00
	Std	1.28E+07	6.12E+09	3.94E+09	1.60E+04	3.04E+08	1.54E+04
F16	Max	1.38E+04	1.31E+04	2.58E+04	8.63E+03	1.18E+04	8.14E+03
	Mean	1.13E+04	1.04E+04	2.02E+04	7.33E+03	8.88E+03	6.47E+03
	Min	7.79E+03	7.60E+03	1.53E+04	6.38E+03	6.77E+03	5.37E+03
	*p*-value	3.69E−11	4.08E−11	3.02E−11	1.25E−05	1.55E−09	1.00E+00
	Std	1.43E+03	1.41E+03	2.65E+03	6.46E+02	1.40E+03	6.95E+02
F17	Max	1.09E+04	1.85E+06	1.03E+07	6.15E+03	8.34E+03	6.16E+03
	Mean	8.03E+03	1.06E+05	2.76E+06	5.29E+03	6.10E+03	5.45E+03
	Min	6.48E+03	5.62E+03	2.66E+05	4.70E+03	4.47E+03	4.56E+03
	*p*-value	3.02E−11	2.15E−10	3.02E−11	2.17E−01	6.67E−03	1.00E+00
	Std	1.16E+03	3.60E+05	2.39E+06	4.29E+02	9.41E+02	5.15E+02
F18	Max	1.39E+07	5.51E+07	4.42E+08	9.87E+06	2.68E+07	3.77E+06
	Mean	6.70E+06	5.47E+06	1.75E+08	3.69E+06	9.98E+06	1.39E+06
	Min	1.46E+06	6.61E+05	6.54E+07	1.39E+06	3.07E+06	4.34E+05
	*p*-value	2.61E−10	4.35E−05	3.02E−11	2.83E−08	4.50E−11	1.00E+00
	Std	3.20E+06	1.08E+07	7.32E+07	2.02E+06	6.29E+06	6.98E+05
F19	Max	1.88E+08	4.48E+09	3.05E+10	1.46E+05	2.53E+08	7.55E+05
	Mean	4.00E+07	8.88E+08	1.89E+10	5.36E+04	1.78E+07	2.06E+05
	Min	1.87E+06	1.91E+07	9.67E+09	9.66E+03	9.77E+05	2.01E+04
	*p*-value	3.02E−11	3.02E−11	3.02E−11	1.29E−06	3.02E−11	1.00E+00
	Std	4.23E+07	1.11E+09	5.30E+09	3.71E+04	4.54E+07	1.80E+05
F20	Max	6.38E+03	7.66E+03	9.29E+03	6.94E+03	7.70E+03	6.06E+03
	Mean	5.65E+03	5.62E+03	8.42E+03	5.63E+03	7.04E+03	5.28E+03
	Min	4.62E+03	4.63E+03	7.26E+03	4.44E+03	6.16E+03	4.15E+03
	*p*-value	3.85E−03	5.55E−02	3.02E−11	4.36E−02	3.02E−11	1.00E+00
	Std	4.39E+02	6.46E+02	5.66E+02	6.36E+02	3.82E+02	4.82E+02
F21	Max	4.41E+03	4.70E+03	5.17E+03	3.37E+03	3.84E+03	3.35E+03
	Mean	3.85E+03	4.13E+03	4.74E+03	3.18E+03	3.39E+03	3.14E+03
	Min	3.45E+03	3.63E+03	4.34E+03	3.01E+03	3.16E+03	2.96E+03
	*p*-value	3.02E−11	3.02E−11	3.02E−11	1.22E−01	2.23E−09	1.00E+00
	Std	1.71E+02	2.28E+02	1.82E+02	1.02E+02	1.52E+02	9.74E+01
F22	Max	2.71E+04	3.46E+04	3.80E+04	3.02E+04	3.39E+04	2.55E+04
	Mean	2.46E+04	2.37E+04	3.59E+04	2.55E+04	2.92E+04	2.23E+04
	Min	2.20E+04	2.02E+04	3.33E+04	2.15E+04	2.37E+04	8.03E+03
	*p*-value	2.49E−06	3.04E−01	3.02E−11	7.60E−07	1.21E−10	1.00E+00
	Std	1.32E+03	3.26E+03	1.34E+03	2.19E+03	2.84E+03	2.90E+03
F23	Max	5.61E+03	6.11E+03	6.30E+03	3.95E+03	4.50E+03	4.37E+03
	Mean	5.04E+03	5.18E+03	5.80E+03	3.78E+03	4.14E+03	3.93E+03
	Min	4.30E+03	4.41E+03	5.30E+03	3.59E+03	3.77E+03	3.67E+03
	*p*-value	3.34E−11	3.02E−11	3.02E−11	1.09E−05	3.83E−06	1.00E+00
	Std	3.59E+02	3.90E+02	2.51E+02	8.41E+01	1.63E+02	1.42E+02
F24	Max	9.17E+03	8.62E+03	9.74E+03	4.90E+03	8.03E+03	5.74E+03
	Mean	7.00E+03	6.85E+03	8.76E+03	4.57E+03	5.99E+03	5.12E+03
	Min	5.33E+03	5.89E+03	7.74E+03	4.38E+03	5.10E+03	4.69E+03
	*p*-value	5.49E−11	3.02E−11	3.02E−11	8.89E−10	1.43E−08	1.00E+00
	Std	7.70E+02	6.40E+02	5.86E+02	1.43E+02	7.07E+02	2.96E+02
F25	Max	8.56E+03	2.65E+04	4.31E+04	6.23E+03	1.21E+04	6.01E+03
	Mean	7.58E+03	1.34E+04	3.41E+04	5.36E+03	8.89E+03	5.00E+03
	Min	6.48E+03	8.74E+03	2.64E+04	4.63E+03	6.37E+03	4.30E+03
	*p*-value	3.02E−11	3.02E−11	3.02E−11	4.71E−04	3.02E−11	1.00E+00
	Std	5.09E+02	4.38E+03	4.47E+03	3.58E+02	1.36E+03	4.21E+02
F26	Max	3.75E+04	5.74E+04	6.64E+04	2.27E+04	3.96E+04	2.97E+04
	Mean	3.26E+04	3.91E+04	5.37E+04	1.94E+04	3.24E+04	2.41E+04
	Min	2.36E+04	2.84E+04	4.61E+04	1.61E+04	2.41E+04	1.01E+04
	*p*-value	4.57E−09	3.34E−11	3.02E−11	6.05E−07	1.55E−09	1.00E+00
	Std	3.49E+03	8.08E+03	4.50E+03	1.65E+03	3.48E+03	4.49E+03
F27	Max	7.14E+03	1.19E+04	1.45E+04	4.73E+03	6.90E+03	5.45E+03
	Mean	5.81E+03	6.07E+03	1.13E+04	4.32E+03	5.30E+03	4.66E+03
	Min	4.81E+03	4.58E+03	7.50E+03	4.01E+03	4.59E+03	4.17E+03
	*p*-value	3.16E−10	1.85E−08	3.02E−11	1.34E−05	2.57E−07	1.00E+00
	Std	5.57E+02	1.47E+03	1.40E+03	1.90E+02	5.30E+02	3.09E+02
F28	Max	1.27E+04	3.61E+04	4.26E+04	9.20E+03	1.43E+04	8.54E+03
	Mean	1.09E+04	1.89E+04	3.65E+04	7.10E+03	1.14E+04	6.37E+03
	Min	8.32E+03	1.35E+04	3.09E+04	5.73E+03	8.32E+03	5.18E+03
	*p*-value	3.34E−11	3.02E−11	3.02E−11	1.04E−04	3.34E−11	1.00E+00
	Std	1.01E+03	5.52E+03	3.40E+03	8.16E+02	1.59E+03	6.56E+02
F29	Max	2.04E+04	1.52E+05	1.68E+06	9.98E+03	1.50E+04	1.09E+04
	Mean	1.57E+04	2.12E+04	4.31E+05	8.90E+03	1.10E+04	9.16E+03
	Min	1.12E+04	1.08E+04	4.78E+04	8.07E+03	7.97E+03	7.69E+03
	*p*-value	3.02E−11	3.34E−11	3.02E−11	1.54E−01	2.20E−07	1.00E+00
	Std	2.28E+03	2.95E+04	4.24E+05	5.65E+02	1.46E+03	8.07E+02
F30	Max	2.78E+09	3.85E+10	4.42E+10	3.04E+07	8.66E+09	1.24E+08
	Mean	1.27E+09	1.32E+10	3.34E+10	1.39E+07	2.19E+09	4.45E+07
	Min	2.53E+08	2.11E+08	1.39E+10	4.87E+06	9.38E+07	7.47E+06
	*p*-value	3.02E−11	3.02E−11	3.02E−11	3.01E−07	1.33E−10	1.00E+00
	Std	6.77E+08	1.34E+10	7.19E+09	7.14E+06	2.88E+09	3.39E+07
